# Evaluation of Fifteen 5,6-Dihydrotetrazolo[1,5-*c*]quinazolines Against *Nakaseomyces glabrata*: Integrating In Vitro Studies, Molecular Docking, QSAR, and In Silico Toxicity Assessments

**DOI:** 10.3390/jof10120816

**Published:** 2024-11-25

**Authors:** Lyudmyla Antypenko, Oleksii Antypenko, Alina Fominichenko, Iryna Karnaukh, Serhii Kovalenko, Mieko Arisawa

**Affiliations:** 1 Independent Researcher, Lamana 11, 69063 Zaporizhzhia, Ukraine; 2 Department of Pharmaceutical, Organic, and Bioorganic Chemistry, Zaporizhzhia State Medical and Pharmaceutical University, M. Prymachenko Ave. 26, 69035 Zaporizhzhia, Ukraine; antypenkoan@gmail.com; 3 Bacteriological Laboratory, Zaporizhzhia Regional Clinical Hospital of Zaporizhzhia Regional Council, Orikhivs’ke Hwy. 10, 69600 Zaporizhzhia, Ukraine; fominichenkoalina@gmail.com (A.F.); irinakarnauh778@gmail.com (I.K.); 4 Research Institute of Chemistry and Geology, Oles Honchar Dnipro National University, Nauky Ave. 72, 49010 Dnipro, Ukraine; kovalenkosergiy@gmail.com; 5 Department of Biosciences and Biotechnologies, Graduate School of Bioresources and Bioenvironment Sciences, Kyushu University, 744 W5-674, Motooka Nishi-ku, Fukuoka 819-0395, Japan; arisawa@agr.kyushu-u.ac.jp

**Keywords:** antifungal activity, *Nakaseomyces glabrata*, 5,6-dihydrotetrazolo[1,5-*c*]quinazolines, molecular docking, toxicity, QSAR

## Abstract

*Nakaseomyces glabrata* (*Candida glabrata*), the second most prevalent Candida pathogen globally, has emerged as a major clinical threat due to its ability to develop high-level azole resistance. In this study, two new 5,6-dihydrotetrazolo[1,5-*c*]quinazoline derivatives (**c11** and **c12**) were synthesized and characterized using IR, LC-MS, ^1^H, and ^13^C NMR spectra. Along with 13 previously reported analogues, these compounds underwent in vitro antifungal testing against clinical *N. glabrata* isolates using a serial dilution method (0.125–64 mg/L). Remarkably, compounds **c5** and **c1** exhibited potent antifungal activity, with minimum inhibitory concentrations of 0.37 μM and 0.47 μM, respectively—about a 20-fold improvement in μM concentration over standard drugs like amphotericin B, caspofungin, and micafungin. A detailed structure–activity relationship analysis revealed crucial molecular features enhancing antifungal potency. Extensive molecular docking studies across 18 protein targets explored potential binding pockets and affinities of the lead compounds. A robust 3D-QSAR model, incorporating molecular descriptors Mor26m and Mor29e, displayed good predictive ability for antifungal activity. In silico predictions indicated an absence of herbicidal effect, negligible environmental toxicity (to honeybees, avian species, and aquatic organisms), and mild human toxicity concerns for these compounds. This comprehensive approach aims to develop novel and effective antifungal compounds against the clinically relevant pathogen *N. glabrata*.

## 1. Introduction

*Nakaseomyces glabrata* (*Candida glabrata*) is a nonhyphae-producing haploid yeast described in 1917 by Harry Warren Anderson as part of the intestinal biota called *Cryptococcus glabratus* [1]. However, it was not until 1995 that Kevin C. Hazen recognized *N. glabrata* as an emerging pathogenic yeast commonly found in patients with diabetes mellitus, solid tumors, malnutrition, in neonates, and sometimes in patients with hematologic neoplasms [2]. Moreover, this haploid yeast species is known for its ability to cause invasive candidiasis [3,4,5,6,7,8,9,10]. Managing *N. glabrata* infections poses significant challenges, as evidenced by data from candidemia cases in Atlanta and Baltimore between 2008 and 2013, where it was the second most prevalent species, accounting for 27% of cases [11]. During this period, there was an increase in multidrug-resistant Candida cases from 1.8% to 2.6%. Similarly, a European study from 2018–2022 across 17 countries showed high proportions of *N. glabrata* (25–33%) in France, the Czech Republic, and the UK [12]. However, the in vitro antifungal susceptibility among the three common Candida species (*C. albicans*, *C. tropicalis*, and *N. glabrata*) obtained before and during the era of COVID-19 did not change significantly [13]. While its colonization was uncommon in community-dwelling individuals, regardless of age, it was much more common in those hospitalized or residing in extended care facilities, additionally in those with dentures [14].

**Figure 1:**
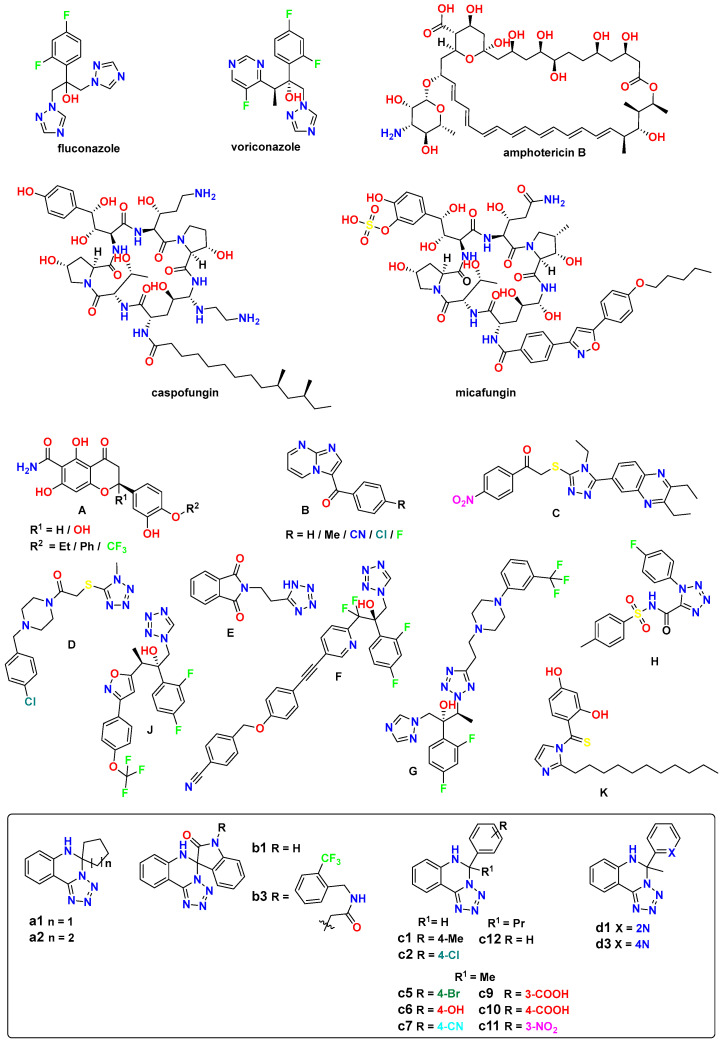
Examples of reported antifungal compounds targeting Candida species, with a focus on the studied 5,6-dihydrotetrazolo[1,5-*c*]quinazolines (numbering follows a previous antimicrobial study [36], and includes investigated substances with two additional novel ones, **c11** and **c12**, for continuity).

Currently used antifungals against *N. glabrata* are azoles (fluconazole, voriconazole), echinocandins (caspofungin, micafungin), and polyenes (amphotericin B) (Figure 1) [3,15,16]. And among the main reasons for their treatment failure are:

1. *Intrinsic Drug Resistance*: natural reduced susceptibility to azoles; innate tolerance to many antifungal compounds; inherently lower sensitivity compared to *C. albicans* [17,18].
2. *Acquired Resistance*: rapid development of resistance during treatment; multiple drug resistance mechanisms, including upregulation of drug efflux pumps (CDR1, CDR2), modifications in drug targets (ERG11 mutations), enhanced stress response mechanisms, and biofilm formation [7,19].
3. *Clinical Challenges*: high mortality rates in systemic infections; limited treatment options; cross-resistance between different drug classes [20,21].

So, the development of novel antifungal compounds has become increasingly imperative due to multiple challenges: the requirement for alternative mechanisms of action, enhanced safety profiles, and improved tissue distribution kinetics [22,23]. This urgency is compounded by the rising incidence of nosocomial fungal infections and an expanding immunocompromised patient population [24,25]. Current therapeutic approaches are compromised by significant treatment failures, resulting in extended hospitalization periods, elevated healthcare costs, and increased mortality rates in cases involving resistant strains [26,27].

Thus, the identification of compounds exhibiting superior potency compared to existing therapeutic agents emphasizes the critical need for innovative antifungal development, particularly in addressing *N. glabrata* infections and emerging antimicrobial resistance patterns.

Recent studies have reported the development of various classes of compounds, that have shown promising antifungal activity against Candida species: hesperetins (**A**), imidazopyrimidines (**B**), quinoxaline-triazoles (**C**), piperazine-tetrazoles (**D**, **G**), tetrazole derivatives (**E**, **H**), pyridine-tetrazoles (**F**), isoxazole-tetrazoles (**J**), and thiobenzoylimidazoles (**K**), etc. (Figure 1). Molecular hybridization has emerged as a promising strategy for developing new antifungal compounds with enhanced activity and selectivity. And by combining two pharmacophore groups or two rings with known activity, synergistic effects can be achieved [28,29,30].

So, hesperetin derivatives **A** are expected to interact in close proximity with the critical active site of the adhesin-like protein AWP1 structure of *N. glabrata* [31,32]. The 3-benzoyl imidazo[1,2-*a*]pyrimidine derivatives **B** were the most active against *Pichia guilliermondii* and *N. glabrata*, indicating an important role in biological activity for the benzene ring with electron-withdrawing substituents [28]. The 2-((5-(2,3-diethylquinoxalin-6-yl)-4-ethyl-4*H*-1,2,4-triazol-3-yl)thio)-1-(4-nitrophenyl)ethan-1-one (**C**) outperformed fluconazole as a control towards the *C. krusei* strain and was at the same level against *N. glabrata* [29].

1-(4-(4-Chlorobenzyl)piperazin-1-yl)-2-((1-methyl-1*H*-tetrazol-5-yl)thio)ethan-1-one **D** was found effective against *C. krusei* and *C. parapsilosis* [30]. 2-(2-(1*H*-Tetrazol-5-yl)ethyl)isoindoline-1,3-dione (**E**) was also among other tetrazole derivatives active against *N. glabrata* [33]. Compound VT-1598 (**F**) effectively controlled in vitro growth of mucosally derived *C. abicans*, *N. glabrata*, *C. utilis,* and *C. krusei* clinical isolates, including fluconazole-resistant strains [10]. Tetrazole derivative **G**, having 3-trifluoromethyl substitution on the phenyl ring of piperazine, was the most active in the series of these compounds against resistant *C. tropicalis* and *C. parapsilosis* [34]. Among the series of 1-phenyl-*N*-tosyl-1*H*-tetrazole-5-carboxamide derivatives, the 4-fluorophenyl substituted one (**H**), in addition to good antibacterial properties, demonstrated strong inhibition of several Candida strains as well as *N. glabrata* [35].

Among the series of (2*R*,3*R*)-3-((3-substituted-phenyl-isoxazol-5-yl)methoxy)-2-(2,4-difluorophenyl)-1-(1*H*-tetrazol-1-yl)butan-2-ol derivatives, compound **J** displayed outstanding antifungal activity against fluconazole-resistant *C. albicans*, *N. glabrata,* and *C. auris* [37]. And 1-(2,4-dihydroxythiobenzoyl)-2-undecyl-imidazole (**K**) was the most active in their group against *N. glabrata* [38].

Hence, the antifungal structure–activity relationship (SAR) on the above-mentioned derivatives had provided some insights into the structural features that were important for their activity:

1. *Heterocyclic ring modifications*. Incorporating heterocyclic rings, such as pyridine, piperazine, triazole, imidazole, or oxazole, in conjunction with the tetrazole moiety, can modulate antifungal activity and selectivity.
2. *Substitution pattern on the tetrazole ring.* Generally, electron-withdrawing substituents such as halogens, trifluoromethyl or nitro groups, on the tetrazole ring or adjacent aromatic rings tends to enhance antifungal activity.
3. *Aryl substituents.* Electron-rich aryl or hetaryl groups are often preferred.
4. *Steric effects*. The introduction of bulky substituents, such as cyclohexyl or benzyl groups, can improve selectivity towards fungal cells over mammalian cells.
5. *Linker chain length and flexibility*. The length and flexibility of the linker chain between the tetrazole moiety and other functional groups can improve the binding affinity to the target enzyme or receptor.
6. *Hydrophobicity and lipophilicity.* Moderate hydrophobicity and lipophilicity of the tetrazole derivatives can enhance their ability to penetrate the fungal cell membrane and reach their target site: long undecyl chains, phenyl rings, etc. However, excessive hydrophobicity or lipophilicity may lead to poor solubility and bioavailability issues.

Moreover, understanding the virulence factors and antifungal resistance mechanisms of *N. glabrata* is crucial for developing effective treatment strategies [39,40,41]. So, the development of azole resistance has been primarily attributed to activating mutations in the pleiotropic drug resistance factor gene *PDR1*, leading to the overexpression of drug efflux pumps such as CDR1, PDH1, and SNQ2 [42,43]. Likewise, deletion of *UPC2A* results in increased susceptibility of *N. glabrata*. Consistently, disruption of Cg*CKB1* and Cg*CKB2* also attenuated the virulence in mouse models of invasive candidiasis [44]. It was demonstrated [45] that a three-helix bundle KIX domain in the Med15a mediator subunit of *N. glabrata* (*Cg*Med15a KIX) plays a crucial role in its growth inhibition by interacting with the PDR1.

Furthermore, other inhibition pathways have been reported, including disruption of ergosterol biosynthesis and cell wall synthesis [10,28,29,30,43,46,47,48,49,50,51,52], targeting adhesin-like proteins [31], serine protease KEX2 [53], fructose-bisphosphate aldolase [54], calcineurin [55], squalene epoxidase [56], histidine kinase [57], proteasome [58], voltage-gated calcium channels [59], heat shock proteins [60], and the non-essential stress kinase YCK2 [61].

These details highlight the importance of carefully optimizing various structural features, physicochemical properties, and pharmacokinetic parameters to develop tetrazole derivatives with potent and selective antifungal activity while maintaining favorable drug-like properties. Overall, fused *N*-heterocyclic ring systems with electron-withdrawing groups, halogen substituents, aryl, or heteroaryl substituents enhance antifungal potency across these diverse molecular scaffolds.

In this context, 5,6-dihydrotetrazolo[1,5-*c*]quinazolines (**a**–**d**, Figure 1) targeting *N. glabrata* is a promising research area. So, in this study, we aim to investigate their in vitro antifungal activity, in silico toxicity, molecular docking, and quantitative structure–activity relationship (QSAR) analysis against *N. glabrata*, providing insights into their potential as effective antifungal agents against this clinically relevant pathogen.

## 2. Materials and Methods

### 2.1. Synthesis

#### 2.1.1. General

Melting points were determined in open capillary tubes in a «Mettler Toledo MP 50» apparatus (Mettler-Toledo, Greifensee, Switzerland), and were uncorrected. The elemental analyses (C, H, N) were performed using the vario EL Cube analyzer (Elementar Americas, Mount Laurel, NJ, USA). Analyses were indicated by symbols of the elements or functions within ±0.3% of the theoretical values. ^1^H NMR spectra (400 MHz) and ^13^C NMR spectra (125 MHz) were recorded on a Varian-Mercury 400 (Varian Inc., Palo Alto, CA, USA) spectrometer with TMS as an internal standard in DMSO-*d_6_* solution. LC-MS was recorded using a chromatography/mass spectrometric system that consists of high-performance liquid chromatography «Agilent 1100 Series» (Agilent, Palo Alto, CA, USA) equipped with a diode-matrix and mass-selective detector «Agilent LC/MSD SL» (atmospheric pressure chemical ionization—APCI). Electron impact mass spectra (EI-MS) were recorded on a Varian 1200 L instrument at 70 eV (Varian, Palo Alto, CA, USA). The purity (>95% pure) of obtained compounds was checked by ^1^H, ^13^C-NMR, and LC-MS. Starting materials and solvents were obtained from Enamine Ltd. (Kyiv, Ukraine) and used without additional purification.

#### 2.1.2. Synthesis of the **c11** and **c12**

2-(1*H*-Tetrazol-5-yl)aniline (1.0 g; 6 mM) was dissolved in propan-2-ol (10 mL). Then, the corresponding aldehyde or ketone (6 mM) was added to the solution, and 1 drop of concentrated sulfuric acid was added. The mixture was refluxed for 1 h. and cooled. A formed precipitate was filtered and washed firstly with propan-2-ol (5 mL) and then with cold water (100 mL). Figures of IR, LC-MS, ^1^H and ^13^C spectra are given in Supplementary Materials.

*5-Methyl-5-(3-nitrophenyl)-5,6-dihydrotetrazolo[1,5-c]quinazoline* (**c11**). Beige solid; 84% yield, mp 233–235 °C. ^1^H NMR (400 MHz): δ (ppm) 8.30 (s, 1H, NH), 8.21 (s, 1H, Ph-2), 8.10 (d, *J* = 8.2 Hz, 1H, Ph-4), 7.77 (d, *J* = 7.5 Hz, 1H, H-10), 7.57 (t, *J* = 8.0 Hz, 1H, Ph-5), 7.43 (d, 1H, d, *J* = 7.9 Hz, 1H, Ph-6), 7.35 (t, *J* = 8.0 Hz, 1H, H-8), 7.09 (d, *J* = 8.2 Hz, 1H, H-7), 6.88 (t, *J* = 7.5 Hz, 1H, H-9), 2.35 (s, 3H, CH_3_). ^13^C NMR (125 MHz): δ (ppm) 149.07, 148.46, 145.02, 142.68, 134.31, 131.72, 131.21, 125.66, 124.19, 120.41, 120.06, 116.16, 107.63, 76.63, 28.51. IR (cm^−1^) 1622, 1525, 1476, 1380, 1340, 1216, 1080, 898, 804, 749, 724, 693. LC-MS: *m*/*z* = 309 [M + H]^+^. Anal. calcd. for C_15_H_12_N_6_O_2_: C, 58.44; H, 3.92; N, 27.26; O, 10.38. Found: C, 58.48; H, 3.87; N, 27.33; O, 10.36.

*5-Phenyl-5-propyl-5,6-dihydrotetrazolo[1,5-c]quinazoline* (**c12**). Beige solid; 48% yield, mp 164–166 °C. ^1^H NMR (400 MHz): 7.88 (s, 1H, NH), 7.72 (d, *J* = 7.7 Hz, 1H, H-10), 7.34–7.13 (m, 5H, Ph), 7.14 (s, 1H), 7.08 (d, *J* = 8.2 Hz, 1H, H-7), 6.81 (t, *J* = 7.5 Hz, 1H, H-8), 2.72 (ddd, *J* = 15.7, 12.0, 4.4 Hz, 1H, CCH_2_), 2.35 (ddd, *J* = 15.0, 12.0, 4.4 Hz, 1H, CCH_2_), 1.77–1.62 (m, 1H, CCH_2_CH_2_), 1.43 (dt, *J* = 12.7, 6.6 Hz, 1H, CCH_2_CH_2_), 1.01 (t, *J* = 7.4 Hz, 3H, CH_3_). IR (cm^−1^): 1622, 1495, 861, 737, 705, 692. LC-MS: *m*/*z* = 292 [M + H]^+^. Anal. calcd. for C_17_H_17_N_5_: C, 70.08; H, 5.88; N, 24.04. Found: C, 70.12; H, 5.84; N, 24.09.

### 2.2. Antifungal Studies

The method of serial dilutions (0.125–64 mg/L and 0.12–62.50 μg/L) of 5,6-dihydrotetrazolo[1,5-*c*]quinazolines (Figure 1, Table 1) on meat-peptone broth was carried out in the bacteriological laboratory of Zaporizhzhia Regional Clinical Hospital of Zaporizhzhia Regional Council (Ukraine) [62] against *Nakaseomyces glabrata* (*Candida glabrata*), *Kluyveromyces marxianus* (*C. kefyr*), and *Cyberlindnera jadinii* (*C. utilis*), which were isolated from patients’ biological material and identified by chromatic Candida media (Liofilchem, Roseto degli Abruzzi, Italy). *C. albicans* ATCC 885-653 was used as a reference strain, provided by Zaporizhzhia City Sanitation Station, Ukraine. Microorganism strains did not reveal sensitivity towards the chosen solvent, namely DMSO (2.5%), that was used to enhance the initial dissolution of the substances. All growth experiments were carried out in duplicate.

The cited procedure [62] is in Ukrainian; therefore, the translation summary is the following: Label sterile 10 mL test tubes from **1** to **8** in one test tube rack and mark it with the corresponding number # of the testing compound. Take the other test tube rack and mark additional test tubes with “*Initial solution of #*”—initial solution of the compound X, sufficient for testing on 5 cultures at the same time; “*Strain*”—culture suspension, diluted 100-fold from the microbiological suspension with standard McFarland turbidity. “*MC*”—media control (meat-peptone broth); “*GC*”—growth control; “*DMSO-GC*”—growth control with 2.5% of DMSO. Shake everything thoroughly after the addition of another component.

Add 9500 μL of broth to the *initial solution of #*. Add 9900 μL of broth to the *strain* tube. Add 2000 μL of broth to the MC tube. Add 1000 μL of broth to tubes **2**–**8** and *GC*. Add 950 μL of broth to the *DMSO-GC* tube. Add 50 μL of *DMSO* to the *DMSO-GC* tube.

*Initial solution of # preparation*: Weigh 0.0512 g of the test compound (if to start testing from 256 mg/L) or 0.0128 g (if to start testing from 64 mg/L), and add 5000 μL DMSO; dissolve thoroughly. Transfer 500 μL of this solution to the *initial solution of #* tube (final quantity of 10 mL is enough for simultaneous determination on 5 cultures to save substance and only use its freshly prepared solutions).

Add 2000 μL of “*Initial solution of #*” to tube **1**. (If determining MIC for 5 cultures, you may add it to each tube, **1** in different racks.) Transfer 1000 μL from tube **1** to tube **2**. Mix thoroughly and transfer 1000 μL to the third tube. Continue dilutions similarly through tube **8**. Remove 1000 μL from tube **8** to maintain a 1 mL final volume.

*Strain suspension preparation*: For each 24 h. culture, begin with its standard McFarland turbidity and perform a 100-fold dilution in isotonic sodium chloride solution. Use the inoculum within 15 min. of preparation.

Add 100 μL of culture suspension (McFarland turbidity 0.5 (1.5 × 10^8^ CFU/mL) for bacteria and 2.0 for Candida strains (6.0 × 10^8^ CFU/mL)) to the *strain* tube already containing broth to obtain a 10 mL solution with 1.5 × 10^6^ CFU/mL for bacteria or 6.0 × 10^6^ CFU/mL for *N. glabrata*.

Add 1000 μL of diluted culture suspension to tubes **1**–**8**, *GC*, *DMSO-GC* (to achieve final concentrations of 7.5 × 10^5^ CFU/mL for bacteria and 3.0 × 10^6^ CFU/mL for *N. glabrata* in a 2 mL mixture).

Incubate at 35 ± 1 °C for 24 h. Examine tubes for visible signs of fungal growth, and compare them to growth controls. Fill the results into a table, where test tubes **1**–**8** correspond to 64–0.125 mg/L dilutions. Repeat everything the other day for experiments to be performed in duplicate.

### 2.3. Molecular Docking Studies

Macromolecule from RCSB Protein Data Bank (PDB) [63] was used as a biological target, namely, metal-binding protein enolase 1 (PDB ID: 7VRD). The 15 mol files of 5,6-dihydrotetrazolo[1,5-*c*]quinazoline derivatives were drawn by MarvinSketch 20.20.0 and saved in mol format; optimized by HyperChem 8.0.8, using the molecular mechanical MM+ algorithm combined with the semiempirical PM3 molecular modeling method with a maximum number of cycles and the Polak–Ribiere (conjugate gradient) algorithm. The next step was a reoptimization of the MM+ optimized structures by applying the semiempirical PM3 molecular modeling method. Obtained files were further used for calculations; mol files were converted to pdb by Open Babel GUI 2.3.2; pdb files were converted to pdbqt by AutoDocTools 1.5.6. Vina 1.1.2 was used to carry out docking studies [64]. The following grid box was used for 7VRD: center_x = −35, center_y = −37, center_z = −4, size_x = 22, size_y = 22, size_z = 22. Discovery Studio v21.1.0.20298 was used for visualization. To validate the docking method by the value of RMSD (root-mean-squared deviation), which characterizes the degree of reliable docking probability, the reference ligand (2-phosphoglyceric acid) was extracted and then reused for the redocking process [65]. If the found pose has an RMSD less than 2 Å relative to the X-ray conformation, then it is generally considered a reasonable docking [66]. So, the RSMD value of 1.124 Å between the experimental and the reference conformation ligand was calculated to be reliable.

Also, CB-Dock2 [67], a protein–ligand auto blind docking tool that inherits the curvature-based cavity detection procedure with AutoDock Vina, was used for calculations of tested substances’ affinity to 18 macromolecules from RCSB Protein Data Bank (PDB) [68] as biological targets, namely, 4N9N, 7VPR, 5TZ1, 5JLC, 4HOG, 7YMU, 1EQP, 7EKU, 7P43, 7O9Q, 4D3W, 7VPS, 2C1T, 4KQ6, 3FWK, 7VRD, 7QP0, and 7VPT online. Vina scores, cavity volumes, docking sizes, and corresponding amino acid contact residues are given in Supplementary Materials, Table S1.

### 2.4. QSAR Modeling

All structures were drawn by MarvinSketch 20.20.0 and saved in mol format, optimized by HyperChem 8.0.8 using the molecular mechanical MM+ algorithm combined with the semiempirical PM3 molecular modeling method with a maximum number of cycles and the Polak–Ribiere (Conjugate Gradient) algorithm. The next step was a reoptimization of the MM+-optimized structures by applying the semiempirical PM3 molecular modeling method. Obtained files were further used for calculations. Descriptors were calculated using Dragon 5.5 (>1500 descriptors) (Dragon 5.5 for Windows, Talete S.r.l., Milano, Italy) by procedure described earlier. Validation of equations in order to confirm their predictive ability was carried out using a prediction set (external) and training set (internal). Cross-validation was performed by the “leave-one-out” method. The optimal equation is one in which the standard error is minimal. The definitions of all used molecular descriptors and the calculation procedures were summarized elsewhere [69,70,71]. The correlation coefficients for all pairs of descriptor variables used in the models were evaluated to identify highly correlated descriptors in order to detect redundancy in the data set. Hence, descriptors with constant variables and near-constant variables were excluded from further consideration (r^2^ ≥ 0.95). The genetic algorithm (GA) and multiple linear regression analysis (MLRA) were used to select the descriptors and to generate the correlation models that relate the structural features to the cell growth percent of different cancer cell lines. The combination of the GA-MLRA technique was applied to obtain the best QSAR models using QSARINS 2.2.4. It splits compound data as follows: random selection of 20% of compounds for the prediction set and 80% for the training set. For each obtained model, such random selection was different. Models that showed statistical significance according to the parameters at a higher level (r^2^ ≥ 0.5) were selected for a more thorough rendering. For these models, the following options were given: the amount of generation algorithm setup was set until seven descriptors, and generation per size was established to the value of 10,000. Parameters of QSAR equation and their definition are given in Supplementary Materials, Table S2.

### 2.5. Toxicity Studies

A tool, CropCSM of Biosig Lab [72,73], was used for online prediction of toxicities of molecules to rapidly identify safe and effective herbicides on honey bee (*A. mellifera*), mallard, and flathead minnow toxicity, in addition to measures of human health, including AMES toxicity, rat LD_50_, and oral chronic toxicity using SMILES of substances (Supplementary Materials, Tables S3 and S4).

## 3. Results and Discussion

### 3.1. Synthesis

The synthetic procedures were reported in the previous study [36], namely the 2-(1*H*-tetrazol-5-yl)aniline undergoes condensation reactions with corresponding aldehydes and ketones under acidic conditions to form a series of substituted 5,6-dihydrotetrazolo[1,5-*c*]quinazolines (**a–d**) (Figure 1).

Among the 15 chosen compounds for investigation, there are two unreported substances: **c11** and **c12**. Hence, LC-MS, elemental analysis, and IR spectra confirmed their structure and the purity. In the ^13^C NMR spectrum of **c11**, the carbon signal of C5 was observed at 76.63 ppm. In the ^1^H NMR spectrum, the signal of quinazoline NH was registered at 8.30 ppm for **c11** and at 7.88 ppm for **c12**; protons of an aromatic ring at 8.21–7.13 ppm, and alkyl substituents at 2.72–1.01 ppm with corresponding multiplicity (Supplementary Materials, Figures of spectra).

### 3.2. Antifungal Studies

Previous computational techniques, such as molecular docking, and absorption, distribution, metabolism, excretion, and toxicity (ADMET) parameters by SwissADME [68,74,75] of **c** series, have provided valuable insights into the binding interactions and pharmacokinetic properties of these tetrazole derivatives, guiding the rational design of potent and selective antimicrobial agents: 4-(5-methyl-5,6-dihydrotetrazolo[1,5-*c*]quinazolin-5-yl)phenol (**c6**) along with corresponding benzoic acid (**c10**) as the most promising molecules for synthesis and drug purposeful search. Moreover, the latter had Vina score stronger than Tedizolid towards ribosomal 50S protein L2P (PDB ID: 2QEX) [68] and to penicillin-binding protein 2X (PDB ID: 2ZC4), additionally with other three substances (**c1**, **c5**, and **c7**) [75]. A search for PAINS (pain-interfering compounds, or frequent-hitting compounds/promiscuous compounds), which are molecules containing substructures that show a strong response in assays independent of the target protein, yielded no hits for all studied compounds [74].

Moreover, preliminary antifungal in vitro studies revealed that the minimum inhibitory concentration (MIC) of **c10** was less than 2 mg/L against *N. glabrata* [76], despite the resistance of *C. kefyr* (*Kluyveromyces marxianus*) and *C. utilis* (*Cyberlindnera jadinii*). And against *C. albicans,* the MIC for **c1** and **c6** was 128 mg/L, and for **c9**, **c10**, and **d1** it was 256 mg/L [36].

So, to obtain valuable results, it was decided to choose 0.125–64 mg/L as the test concentration range against *N. glabrata*. Summing up the serial dilution method (Section 2), 0.0128 g of the test substance was dissolved in 5 mL of DMSO. Subsequently, 0.5 mL of this solution was transferred and diluted with 9.5 mL of meat-peptone broth. For the first test tube, 1 mL of this dilution was combined with 1 mL of *N. glabrata* suspension (6.0 × 10^6^ CFU/mL), resulting in a final volume of 2 mL with a test substance concentration of 64 mg/L, DMSO concentration of 2.5%, and *N. glabrata* suspension of 3.0 × 10^6^ CFU/mL. Serial two-fold dilutions were performed for subsequent test tubes, thereby reducing both the test substance and DMSO concentrations proportionally. Microorganism strains did not reveal sensitivity towards the chosen solvent, namely DMSO, that was used to enhance the initial dissolution of the substances. According to Liu et al. [77], when conducting drug susceptibility testing for *N. glabrata*, the maximum permissible concentrations for common organic solvents are as follows: DMSO and acetone should not exceed 2.5%, while ethanol and methanol should remain below 5%. All growth experiments were carried out in duplicate.

**Figure 2:**
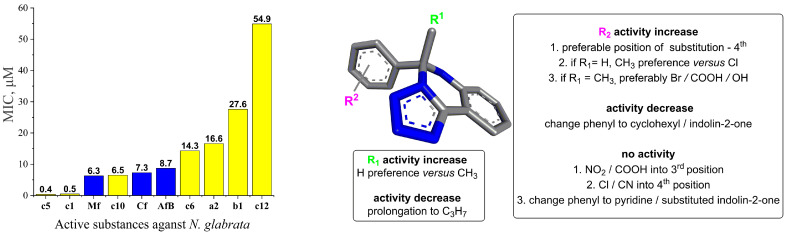
Minimum inhibitory concentration (μM) of 5,6-dihydrotetrazolo[1,5-*c*]quinazolines (yellow color) and references (**Mf**: micafungin, **Cf**: caspofungin, **AfB**: amphotericin B; blue color). And their structure–activity relationship against *N. glabrata.* General molecular structure was optimized by HyperChem 8.0.8, and Discovery Studio v21.1.0.20298 was used for 3D visualization.

In the result, half of the studied tetrazole derivatives exhibited varying degrees of antifungal inhibition properties (Table 1, Figure 2). Notably, compound **c12** showed inhibition at a concentration of 16 mg/L, while compounds **b1**, **c6**, and **a2** displayed inhibition at 8 mg/L, 4 mg/L, and 4 mg/L, respectively. Compound **c10** exhibited inhibition at a concentration as low as 2 mg/L.

The most potent compounds were **c1** and **c5**, which demonstrated inhibition at the remarkably low concentration of 0.125 mg/L, which is 64-fold lower than the MIC of 8 mg/L for amphotericin B and caspofungin, while micafungin showed inhibition at 4 mg/L. Due to their exceptional potency, compounds **c1** and **c5** were further studied by diluting them ten-fold (0.12–62.50 μg/L), but fungal growth was observed, indicating their MICs were not lower. Also, it is interesting that compounds **c9** and **d1** were not active against *N. glabrata* as earlier against *C. albicans* [36]. And substance **c10** was two times more active than micafungin, and **a2** with **c6** was at the same level but of higher concentration in μM.

Furthermore, the antifungal activity results revealed that the solubility profile of the compounds likely did not play a significant role in the observed potency. Notably, compounds **a1**, **b1**, **c1**, **c2**, **c5**, **c7**, **c9**, **c11**, **c12**, **d1,** and **d3** were soluble in propan-2-ol only during boiling conditions, while the other compounds were soluble in propan-2-ol at r.t. However, none of the compounds demonstrated appreciable water solubility. So, the use of DMSO allowed for the preparation of the stock solutions that could be further diluted to the desired test concentrations (initially 2.5% for 64 mg/L). This approach ensured that any observed antifungal activity could be more directly attributed to the intrinsic potency of the compounds, rather than being limited by poor solubility or the effect of DMSO.

Still, limited aqueous solubility of some of the compounds may have hindered their ability to effectively penetrate the fungal cell membrane and reach the intended intracellular targets (e.g., antifungal results of **c9** vs. **c10**). Also, lack of selectivity and/or binding interactions towards the key fungal targets may have contributed to their reduced antifungal potency.

### 3.3. Structure–Activity Relationship

Based on the obtained antifungal activity results, SAR against *N. glabrata* can be summarized as follows (Figure 2).

1. R^1^ alkyl prolongation: extending the alkyl chain from methyl to propyl may introduce unfavorable steric clashes or conformational restrictions, leading to decreased activity.
2. 4th Position substitution of phenyl ring: the preference for substitution at the 4th position over other positions on the bicyclic ring system suggests that the steric and electronic environment at this specific site is optimal for binding to the target enzyme. Substituents at this position may participate in critical interactions like hydrogen bonding, π-stacking, or filling a hydrophobic pocket. Besides the presence of an aromatic moiety in these compounds, increased hydrophobicity, which improves their permeability into the cell membrane, therefore enhances the antifungal activity.
3. CH_3_ vs. Cl when R^1^ = H: the preference for a methyl group over chloro, when R^1^ is unsubstituted, could be attributed to the more lipophilic nature of the methyl substituent and to steric factors, where the smaller hydrogen atom allows for better accommodation and binding within the target pocket. The chloro group, being larger and more electronegative, may experience unfavorable steric clashes or result in suboptimal binding interactions.
4. Br/COOH/OH vs. CN when R^1^ = methyl: the preference for bromo, carboxyl, or hydroxyl substituents over a cyano group at R^2^ suggests that the electron-withdrawing nature of the groups may be disfavored. The electron-rich bromine, carboxyl, and hydroxyl groups could form favorable hydrogen bonding or ionic interactions with the target.
5. Change from phenyl to cyclohexyl or indolin-2-one substituents: this structural modification leads to a decrease in the biological activity, potentially indicating that the size of the rings is important for the desired activity.
6. NO_2_/COOH substitution into the 3rd position: the introduction of strongly electron-withdrawing nitro or carboxyl groups at the 3rd position may significantly alter the electronic distribution and potentially disrupt crucial binding interactions, leading to a complete loss of activity.
7. Cl substitution into the 4th position: Similar to the 3rd position substitution, placing a chloro group at the 4th position of the R^2^ phenyl ring also leads to a complete lack of activity. This indicates that the specific substitution pattern on the phenyl ring is essential for the compound to exhibit the desired antifungal effects.
8. Change from phenyl to pyridine or substituted indolin-2-one: similar to the cyclohexyl and indolin-2-one modifications, changing the phenyl ring to a pyridine or substituted indolin-2-one moiety likely disrupts essential aromatic interactions or introduces steric hindrances, leading to a complete loss of activity.

In summary, the SAR analysis suggests that the R^2^ substituent plays a critical role in maintaining the desired biological activity. Changing the phenyl ring to a cyclohexyl or indolin-2-one moiety likely disrupts crucial π-π stacking or aromatic interactions with the target binding site, resulting in decreased activity.

### 3.4. Molecular Docking

Using computational methods like molecular docking [78], it may be possible to explore binding modes and identify other substituents or scaffolds that can occupy different pockets within the enzyme active site. These in silico predictions can guide the design and synthesis of novel compounds to achieve better fit and higher binding affinity.

Table 2 presents the results of online molecular docking calculations by the CB-Dock2 website [67,79] for two lead compounds, **c1** and **c5**, against 18 various antifungal protein targets (from RCSB Protein Data Bank (RCSB PDB) [63]) taken majorly from *N. glabrata*, *Saccharomyces cerevisiae*, and *C. albicans*.

**Table 2:** The strongest calculated affinity to the various antifungal targets of **c1** and **c5**, kcal/mol.

#	Strain *	Classification	Molecule ID	PDB ID **	#	Vina Score
1	SC S288C	transcription	sterol uptake control protein 2	4N9N	**c1**	−9.6
					**c5**	−9.9
2	NG CBS138	transcription	sterol uptake control protein 2	7VPR	**c1**	−10.4
					**c5**	−7.9
3	CA	oxidoreductase/ oxidoreductase inhibitor	sterol 14-alpha demethylase	5TZ1	**c1**	−10.2
					**c5**	−9.2
4	NG CBS138	oxidoreductase/ oxidoreductase inhibitor	lanosterol 14-alpha demethylase	5JLC	**c1**	−9.6
					**c5**	−8.8
5	NG CBS138	oxidoreductase/ oxidoreductase inhibitor	dihydrofolate reductase	4HOG	**c1**	−8.5
					**c5**	−7.9
6	NG	oxidoreductase/ oxidoreductase inhibitor	NADPH-dependent methylglyoxal reductase GRE2	7YMU	**c1**	−8.1
					**c5**	−8.2
7	CA	hydrolase	exo-beta-(1,3)-glucanase	1EQP	**c1**	−9.6
					**c5**	−9.7
8	NG CBS138	Sugar-binding protein	4-alpha-glucanotransferase	7EKU	**c1**	−9.5
					**c5**	−9.2
9	NG CBS138	carbohydrate	1,4-alpha-glucan-branching enzyme	7P43	**c1**	−9.3
					**c5**	−8.8
10	NG CBS138	cell adhesion	adhesin-like wall protein 1 A-domain	7O9Q	**c1**	−9.4
					**c5**	−9.1
11	NG CBS 138	cell adhesion	epithelial adhesin 1	4D3W	**c1**	−7.1
					**c5**	−7.4
12	NG CBS138	protein transport	importin subunit alpha	7VPS	**c1**	−9.8
					**c5**	−9.3
13	SC	protein transport	importin alpha subunit	2C1T	**c1**	−9.2
					**c5**	−8.7
14	NG CBS138	transferase	6,7-dimethyl-8-ribityllumazine synthase	4KQ6	**c1**	−9.4
					**c5**	−9.4
15	NG	transferase	flavin mononucleotide adenylyltransferase	3FWK	**c1**	−7.9
					**c5**	−7.9
16	CA SC5314	Metal-binding protein	enolase 1	7VRD	**c1**	−8.1
					**c5**	−8.1
17	NG	apoptosis	metacaspase-1	7QP0	**c1**	−7.6
					**c5**	−7.8
18	NG CBS 138	protein transport	importin alpha arm domain	7VPT	**c1**	−7.1
					**c5**	−7.0

* SC—Saccharomyces cerevisiae, NG—Nakaseomyces glabrata, CA—Candida albicans. ** Protein targets are taken from RCSB Protein Data Bank [79].

It is worth mentioning that *N. glabrata* shares a recent common ancestor with several Saccharomyces species and belongs to a clade different from that of other Candida species (namely those that recode the *CUG* codon to serine) [80]. Vina scores of the strongest affinities, cavity volumes, docking sizes, and corresponding amino acid contact residues are presented in Supplementary Materials, Table S1.

The docking results indicate that both compounds **c1** and **c5** have the potential to interact with a diverse range of protein targets involved in various cellular processes in Candida and Saccharomyces species. The strongest predicted binding affinity (kcal/mol) was observed for **c1** against importin subunit alpha (−9.8), sterol 14-alpha demethylase (−10.2), and sterol uptake control protein 2 (−10.4) (Table 2). While compound **c5** showed the highest docking scores against 6,7-dimethyl-8-ribityllumazine synthase (−9.4), exo-*β*-(1,3)-glucanase (−9.7), and also sterol uptake control protein 2 (−9.9).

The strong predicted binding to key targets such as sterol biosynthesis enzymes, transcription factors, and cell wall-modifying enzymes suggests that these compounds could be explored further in vitro against them as potential antifungal agents. Nevertheless, both compounds exhibited relatively lower docking scores against dihydrofolate or methylglyoxal reductase and flavin mononucleotide adenylyltransferase, suggesting potentially weaker binding to these proteins.

**Figure 3:**
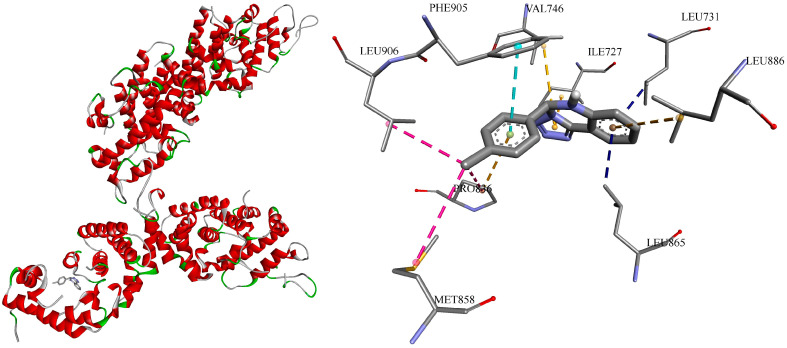
Visual 3D representation of the sterol uptake control protein 2 (PDB ID: 7VPR) with lead-compound **c1** (Vina score −10.4 kkal/mol), showing bonds formation in its cavity of chain D. All ten formed bonds were hydrophobic: π-σ in blue color; π-π stacked in light blue color; alkyl in pink color; π-alkyl in orange color. Discovery Studio v21.1.0.20298 was used for 3D visualization.

To inspect the binding poses and understand the interactions between the ligand and receptor, it was decided to 3D visualize the highest affinity result (−10.4 kcal/mol), namely, **c1** towards sterol uptake control protein 2 (PDB ID: 7VPR) (Figure 3).

Hence, all 10 formed bonds (amino acid/distance in Ǻ) of **c1** were hydrophobic: π-σ (LEU731/3.51, LEU865/3.48); π-π stacked (PHE905/4.40); alkyl (PRO836/4.52, MET858/5.00, LEU906/4.38); and π-alkyl (LEU886/5.28, ILE727/4.89, VAL746/4.77, PRO836/4.59), showing the structure’s flexibility to fit into the cavity of protein D chain.

It is interesting and worth mentioning that in the previous study [36], the docking grid was centered at (71, 66, 4) with dimensions (14, 16, 14) into sterol 14-alpha demethylase by obtained X-ray results (PDB ID: 5TZ1), and AutoDock Vina scores for **c1** and **c5** were −8.2 and −8.3 kcal/mol. While now CB-Dock2 [67] identified a different best binding pocket for them centered at (66, 35, 41) with dimensions (32, 28, 19) with the highest predicted affinities of −10.2 and −9.2 kcal/mol, respectively (Supplementary Materials, Table S1).

Whereas, among 5 other cavities proposed by CB-Dock2 on 5TZ1, one was found with the same X-ray-chosen coordinates, and affinity for **c1** and **c5** in this pocket was a bit stronger: −8.8 and −8.9 kcal/mol, respectively.

So, we decided to perform one more additional molecular docking calculation to see what the difference will be in the grid and affinity scores on one more protein. Considering the same found in vitro MIC of **c1** and **c5,** we have chosen metal-binding protein enolase 1 (PDB ID: 7VRD), because their Vina scores were also predicted as being the same (−8.1 kcal/mol) by CB-Dock2 [67] (Table 3). Calculated RMSD (root mean square deviation) was obtained as 1.124 Å, so results were considered reliable [65,66].

The docking grid centered at (−35, −37, 4) with dimensions (22, 22, 22) was found according to the position of the reported ligand, and the affinity of **c1** and **c5** was calculated to be −7.8 kcal/mol off-site by AutoDock Vina. While CB-Dock2 proposed a different best binding pocket, centered at (−10, −18, 24) with dimensions (35, 31, 35). And the predicted score for each compound was −8.1 kcal/mol. Nevertheless, a second-best potential binding pocket by CB-Dock2 was proposed with the same coordinates as the reported X-ray grid, and affinity for **c1** and **c5** in this pocket was practically the same: −7.7 and −7.8 kcal/mol, as shown by us, respectively.

Hence, online tools like CB-Dock2 can facilitate and guide enzymatic studies of potential biologically active compounds. And this multi-pronged strategy, integrating both in vitro and further in vivo studies with structural and mechanistic characterization, can provide a high level of confidence in the substance’s target-specific reactivity and its potential for development as a selective modulator of the enzyme of interest.

### 3.5. Quantitative Structure–Activity Relationship

**Figure 4:**
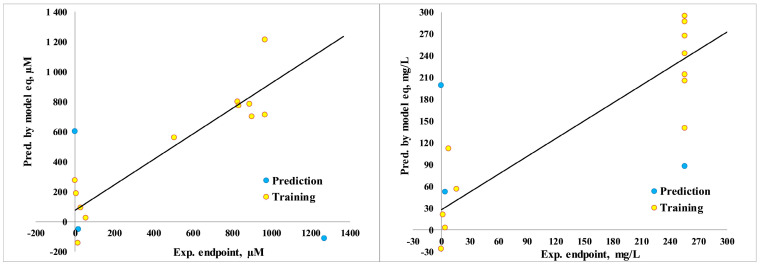
Correlation graphs of predicted vs. experimental MIC (μM/mg/L) of model equations.

Furthermore, the synergistic blend of organic synthesis, analytical chemistry, pharmaceutical chemistry, and molecular docking within the framework of the quantitative structure–activity relationship (QSAR) significantly contributes to the advancement of novel antifungal drug development [81,82,83]. This integrative approach can help predict the biological activity of new tetrazole derivatives, guiding the design of more potent antifungal agents. Thus, the calculated QSAR models of antifungal activity showed a high goodness-of-fit (r^2^ = 0.81–0.85, Figure 4) and predictive ability (Q^2^loo = 0.68–0.71), indicating its reliability in modeling.

*Model-1*, μM = −860.236(±487.862) × Mor26m + 3002.6084 (±1007.9199) × Mor29e + 1374.0919 (±311.2194). n = 15, r^2^ = 0.8474; s = 189.6460; F = 24.9931; *p* = 0.0001; RMSEtr = 164.2383; R^2^cv (Q^2^loo) = 0.6781; R^2^-R^2^cv = 0.1694; RMSEcv = 238.5710; MAEcv = 198.0963; PRESScv = 682,993.3683; CCCcv = 0.8349; RMSEex = 871.0944; MAEex = 683.7867; PRESSext = 2,276,416.4022.

where Mor26m and Mor29e: 3D-MoRSE descriptors, weighted by mass and Sanderson electronegativity (Supplementary Materials, Table S2) [69].

*Model-2*, mg/L = 158.3513 (±95.4438) × Mor10m + 871.3969 (±329.9217) × Mor29e + 294.6011 (±75.7156). n = 15, r^2^ = 0.8142; s = 61.3710; F = 19.7157; *p* = 0.0001; RMSEtr = 53.1489; R^2^cv (Q^2^loo) = 0.7114; R^2^-R^2^cv = 0.1028; RMSEcv = 66.2355; MAEcv = 53.7468; PRESScv = 52,645.7556; CCCcv = 0.8516; RMSEex = 153.0649; MAEex = 138.7554; PRESSext = 70,286.6325.

So, the significance of the models is supported by the low *p*-value of the F-statistic and low error metrics [70]. However, using only 15 data points for model validation can be a limitation, especially in the context of QSAR modeling, where having a larger dataset can improve the robustness and reliability of the model in future studies. Nevertheless, it was found that 3D-MoRSE descriptors, Mor26m and Mor29e for mass and Sanderson electronegativity, were important for inhibiting the *N. glabrata* pathway.

### 3.6. Toxicity Prediction

**Figure 5:**
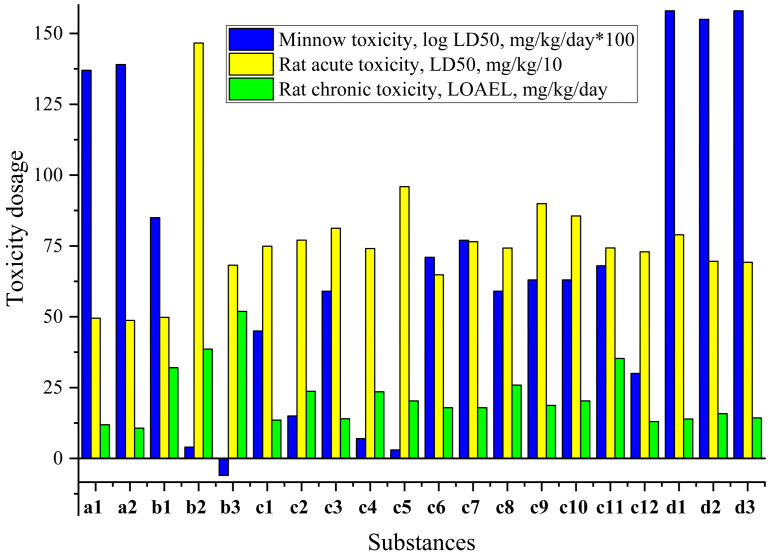
Calculated minnow toxicity (log LD_50_, mg/kg/day, results were multiplied in 100 for the same scale; results below 30: high acute), rat acute toxicity (LD_50_, mg/kg; results were divided in 10 for the same scale; results under 5: strong; 5–50: moderate; 50–500: slightly; over 500: safe), and rat chronic toxicity (lowest observed adverse effect level (LOAEL), mg/kg/day; results under 10: strong; 10–50: medium; over 50: weak).

Furthermore, pharmaceuticals and agrochemicals have been linked to various undesirable negative impacts on health and the environment. To aid in identifying green fungicides, the cropCSM [72,73] provides an assessment of a molecule’s impact on honey bee (*A. mellifera*) toxicity, as well as toxicity to mallards and flathead minnows (Figure 5). Additionally, it includes measures of human health, such as AMES toxicity, rat LD_50_, and oral chronic toxicity (Supplementary Materials, Table S4). Hence, considering environmental toxicity, all the compounds show no herbicidal and no honey bee toxicity. Only compounds **a1** and **a2** show avian toxicity, while the rest of the compounds do not. The aquatic minnow toxicity values (log LD_50_, mg/kg/day) range from −0.06 to 1.58, not of dangerous level. The presence of the nitro group may contribute to the increased minnow toxicity, as nitro groups are electron-withdrawing and increase the reactivity of the compounds.

Mentioning human toxicity, namely, AMES toxicity: most of the compounds (except **b2** and **b3**) show positive results, due to some functional groups that might influence the mutagenic potential of compounds. Nevertheless, the rat acute toxicity (LD_50_, mg/kg) values range from 487.1 to 1465.9 (Supplementary Materials, Table S4) of the light level. The majority of rat chronic toxicity (LOAEL, mg/kg/day) values range from 10.7 to 51.9, also indicating medium toxicity.

So, from the provided data, it appears that the compounds exhibit varying levels of toxicity across different endpoints, still of moderate effect.

### 3.7. Pearson Correlations

**Figure 6:**
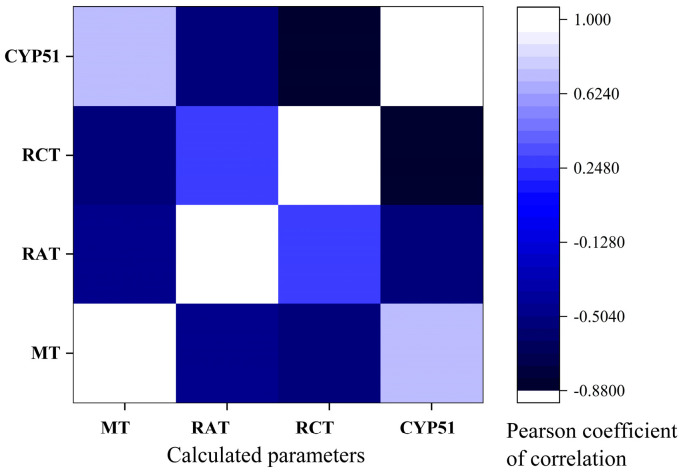
Pearson coefficient of correlation between predicted affinity (Vina score, kcal/mol) to CYP51 (sterol 14-alpha demethylase, PDB ID: 5TZ1) [36] and toxicity (MT: minnow toxicity, log LD_50_, mg/kg/day), RAT: rat acute toxicity (LD_50_, mg/kg), RCT: rat chronic toxicity (LOAEL, mg/kg/day) [72].

By analyzing the relationships between the predicted toxicity, in silico antifungal affinity, and found in vitro activities, it may be possible to identify key structural determinants that contribute to the observed toxicological profiles. And this information could be valuable for the design and development of new compounds with improved safety profiles or for the optimization of existing compounds to mitigate potential adverse effects. So, based on the Pearson correlation results presented in Figure 6 (Supplementary Materials, Table S5), the following conclusions are found.

Minnow toxicity (MT) has a strong positive correlation (r^2^ = 0.72534) with CYP51 (sterol 14-alpha demethylase, PDB ID: 5TZ1) affinity [36]. This suggests that compounds with lower affinity for the CYP51 enzyme tend to have lower minnow toxicity. It could be caused by the formation of fewer bonds with proteins, so it is less toxic. MT has a moderate negative correlation with rat acute toxicity (RAT) (r^2^ = −0.48236) and rat chronic toxicity (RCT) (r^2^ = −0.55494). This implies that compounds with lower minnow toxicity tend to have higher acute and chronic toxicity in rats due to different mechanisms of action. RCT has a strong negative correlation with CYP51 affinity (r^2^ = −0.87526), suggesting that compounds with higher rat chronic toxicity tend to have higher affinity for the CYP51 enzyme.

And there was found to be no statistically significant correlation of predicted toxicities with MIC (Supplementary Materials, Table S5). It is important to note that correlation does not necessarily imply causation, and further experimental validation and mechanistic studies may be needed to confirm these relationships and understand the underlying biological mechanisms.

### 3.8. Limitations of Study

1. *Limited sample size for QSAR modeling*: the QSAR models were developed using only 15 data points, which is a relatively small sample size. A larger dataset would improve the robustness and reliability of the QSAR models.
2. *Lack of experimental validation*: the study heavily relies on computational techniques like molecular docking and QSAR analysis but lacks extensive experimental validation of the predicted antifungal activities and binding interactions.
3. *Narrow focus on N. glabrata*: the study is primarily focused on *N. glabrata*, while other clinically relevant Candida species are not extensively investigated. Expanding the scope to include a broader range of fungal pathogens would provide a more comprehensive understanding.
4. *Limited assessment of toxicity*: the toxicity predictions are based on in silico tools, and the study does not report any experimental toxicological data. Further in vitro and in vivo toxicity assessments are necessary to fully evaluate the safety profiles of the compounds.
5. *Lack of mechanistic studies*: the study does not delve deeply into the underlying molecular mechanisms of action for the antifungal activities of the compounds. More detailed mechanistic investigations, such as in vitro target identification and validation, would strengthen the understanding of the structure–activity relationships.

### 3.9. Strengths of Study

1. *Comprehensive literature review*: the manuscript provides a summary of the most relevant literature on *N. glabrata* studies, antifungal resistance, and the development of various classes of antifungal compounds, targeted on heterocyclic azo ones.
2. *Systematic SAR analysis*: the study presents a detailed SAR analysis, which highlights the importance of specific structural features for the antifungal activity against *N. glabrata.*
3. *Utilization of advanced computational techniques*: a range of computational approaches are effectively employed, including molecular docking, QSAR modeling, and in silico toxicity predictions, to guide the drug discovery process.
4. *Identification of potent lead compounds*: the study identified two highly potent compounds, **c1** and **c5**, which exhibited remarkable antifungal activity against *N. glabrata* at remarkably low concentrations.
5. *Exploration of diverse antifungal targets*: the molecular docking analysis investigated the potential binding of the compounds to a wide array of protein targets, suggesting a multi-targeted antifungal mechanism of action.
6. *Reporting of novel compounds*: the manuscript reports the synthesis and characterization of two previously unreported 5,6-dihydrotetrazolo[1,5-*c*]quinazoline derivatives (**c11** and **c12**).
7. *Detailed structural data and visualizations*: the manuscript provides detailed structural information, including 3D visualizations of the predicted binding poses, which enhance the understanding of the structure–activity relationships.
8. *Acknowledgment of limitations and future directions*: the manuscript acknowledges the limitations of the study and outlines clear future research directions to address them, demonstrating a thoughtful and rigorous approach.

### 3.10. Future Directions

Experimental studies, such as in vitro enzyme inhibition assays, cell-based and biophysical binding assays, should be conducted to confirm the in silico predicted interactions and evaluate the toxicity of the tested substances as well as to assess the specificity and selectivity of the compounds towards their intended targets, as well as potential off-target effects. It is important to do thorough research on the mechanisms of action, considering how drugs affect gene expression, cellular pathways, and phenotypic alterations.

Promising lead compounds should be evaluated for their in vivo antifungal efficacy, safety, and pharmacokinetic profiles in appropriate animal models of fungal infections.

Improving the overall solubility profiles, particularly in physiologically relevant solvents, may be an important factor to consider in future optimization efforts for this series of tetrazole-based compounds. Strategies, such as structural modifications, formulation approaches, or the use of alternative solubilizing agents, could be explored to unlock the full potential of the more potent but less soluble analogues.

Furthermore, investigating the possible synergistic effects of these substances with already available antifungal drugs or other relevant compounds may result in the development of more potent and comprehensive antifungal treatments.

## 4. Conclusions

Overall, the increasing prevalence of *N. glabrata* infections and the associated challenges of antifungal resistance have catalyzed extensive research efforts to discover novel antifungal substances. 5,6-Dihydrotetrazolo[1,5-*c*]quinazolines have emerged as promising candidates, exhibiting potent antifungal activities against *N. glabrata* through various predicted mechanisms of action. Notably, compounds **c1** and **c5** demonstrated remarkable inhibition at concentrations as low as 0.125 mg/L, outperforming reference drugs like amphotericin B, caspofungin and micafungin. The SAR analysis provided insights into the structural features essential for antifungal activity: the presence of heterocyclic rings, bulky aryl, or heteroaryl groups was found to enhance hydrophobic interactions, and electron-rich bromine, carboxyl, and hydroxyl groups could form favorable hydrogen bonding or ionic interactions with the target. Computational docking studies predicted strong binding affinities of compounds **c1** and **c5** towards various antifungal targets, including sterol biosynthesis enzymes, transcription factors, protein transport, and cell wall-modifying enzymes in *N. glabrata* and related species. QSAR models were developed, demonstrating good predictive ability and identifying 3D-MoRSE descriptors related to mass and electronegativity as important for inhibiting fungal growth. In silico toxicity predictions suggested low to moderate toxicity levels for most compounds, with varying profiles across different endpoints.

Hence, the integration of synthetic chemistry, molecular hybridization strategies, computational techniques, along with further in vitro and in vivo experimental validations, and the exploration of alternative targets has paved the way for the development of more effective and selective antifungal agents against *N. glabrata*.

## Figures and Tables

**Table 1:** Antifungal activity results against *N. glabrata* by in vitro serial dilution method.

Substance	Minimum Inhibitory Concentration (64–0.125 mg/L), Concentration of Substance (μM)									
	64	32	16	8	4	2	1	0.50	0.25	0.125
**c1**	− *	−	−	−	−	−	−	−	−	0.47
**c5**	−	−	−	−	−	−	−	−	−	0.37
**c10**	−	−	−	−	−	6.50	+	+	+	+
**a2**	−	−	−	−	16.58	+	+	+	+	+
**c6**	−	−	−	−	14.32	+	+	+	+	+
**b1**	−	−	−	27.56	+	+	+	+	+	+
**c12**	−	−	54.91	+	+	+	+	+	+	+
Growth control	+	+	+	+	+	+	+	+	+	+
2.5% DMSO control	+	+	+	+	+	+	+	+	+	+
Sterility control	−	−	−	−	−	−	−	−	−	−

* Absence (−)/presence (+) of opalescence. Minimum inhibitory concentration of references: amphotericin B: 8 mg/L (8.66 μM), caspofungin: 8 mg/L (7.32 μM), and micafungin: 4 mg/L (6.30 μM). **a1, b3, c2, c7, c9, c11, d1, d3**: no activity. Repeated twice.

**Table 3:** Affinity of investigated substances towards to enolase 1 (PDB ID: 7VRD), kcal/mol.

Substance/Affinity to Enolase 1, kcal/mol *														
b3	b1	c7	c10	c9	d1	c11	c5	c1	c2	a2	c6	a1	d3	c12
−10.3	−8.5	−8.2	−8.2	−8.0	−7.9	−7.8	−7.8	−7.8	−7.8	−7.7	−7.6	−7.4	−7.4	−7.1

* Calculated RMSD: 1.124 Å [65,66].

## Data Availability

The original contributions presented in the study are included in the article/Supplementary Material, further inquiries can be directed to the corresponding author.

## Supplementary Materials

The following supporting information can be downloaded at: https://www.mdpi.com/article/10.3390/jof10120816/s1, Figures of IR, LC-MS, ^1^H and ^13^C spectra of **c11** and **c12**; Table S1: CB-Dock2 website results of cavity detection and protein–ligand blind molecular docking. Table S2: Parameters of QSAR equation and their definition; Table S3: SMILES of studied substances; Table S4: Predicted herbicide, environmental and human toxicity by CropCSM of Biosig Lab; Table S5: Pearson correlation results calculated in Origin 2018.
