# Supplementary material for: Evaluation of Fifteen 5,6-Dihydrotetrazolo[1,5-c]quinazolines Against Nakaseomyces glabrata: Integrating In Vitro Studies, Molecular Docking, QSAR, and In Silico Toxicity Assessments
Source: J Fungi (Basel). 2024 Nov 25;10(12):816. doi: 10.3390/jof10120816 (PMC11728297; doi:10.3390/jof10120816)
Supplement: Supplementary file 1 [file jof-10-00816-s001.zip › jof-3302148-supplementary.pdf]

Supplementary materials

Evaluation of Fifteen 5,6-Dihydrotetrazolo[1,5-*c*]quinazolines Against *Nakaseomyces glabrata*: Integrating *In Vitro* Studies, Molecular Docking, QSAR, and *In Silico* Toxicity Assessments

Lyudmyla Antypenko[1*], Oleksii Antypenko[2], Alina Fominichenko[3], Iryna Karnaukh[3], Serhii Kovalenko[4], Mieko Arisawa[5]

[1] Independent Researcher, Lamana 11, 69063 Zaporizhzhia, Ukraine

[2] Department of Pharmaceutical, Organic, and Bioorganic Chemistry, Zaporizhzhia State Medical and Pharmaceutical University, M. Prymachenko Ave. 26, 69035 Zaporizhzhia, Ukraine

[3] Bacteriological Laboratory, Zaporizhzhia Regional Clinical Hospital of Zaporizhzhia Regional Council, Orikhivs'ke Hwy. 10, 69600 Zaporizhzhia, Ukraine

[4] Research Institute of Chemistry and Geology, Oles Honchar Dnipro National University, Nauky Ave. 72, 49010 Dnipro, Ukraine

[5] Department of Biosciences and Biotechnologies, Graduate School of Bioresources and Bioenvironment Sciences, Kyushu University, 744 W5-674, Motooka Nishi-ku, Fukuoka 819-0395, Japan

*Corresponding author.
E-mail address: antypenkol@gmail.com

Figures of IR, LC-MS, ^{1}H and ^{13}C spectra of **c11** and **c12** spectra.

IR spectrum of **c11**.

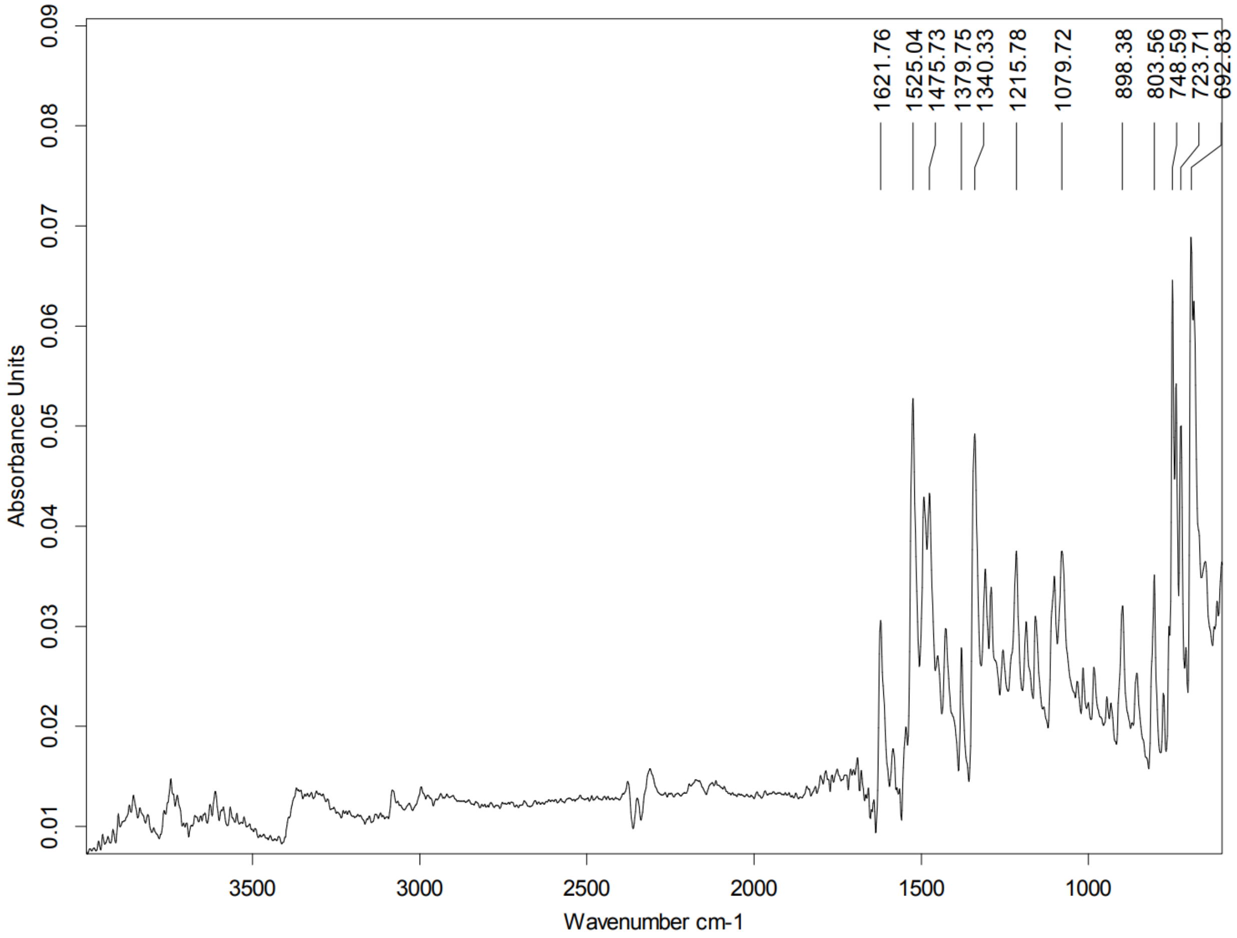

LC-MS spectrum of **c11**.

MaxPeak: 100.00%
Ret_Time: 1.270 min

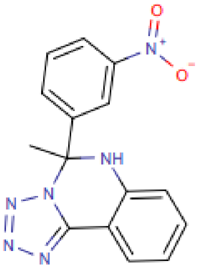

Mol Wt 308.3

Exact Mass 308.1

#	Time	Area%
1	1.270	100.00

RT 1.288

RT 1.288

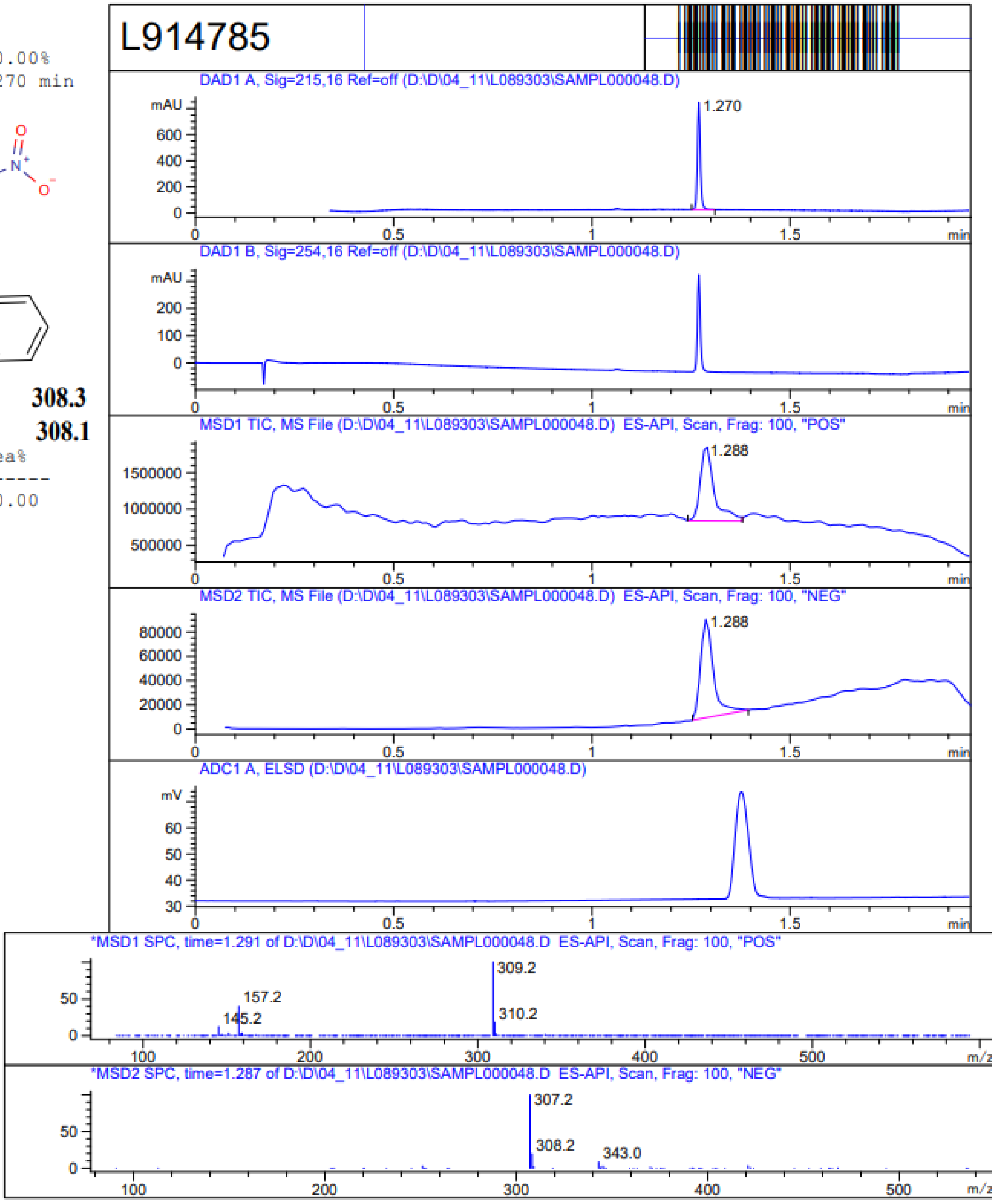

^{1}H and ^{13}C spectra of **c11**.

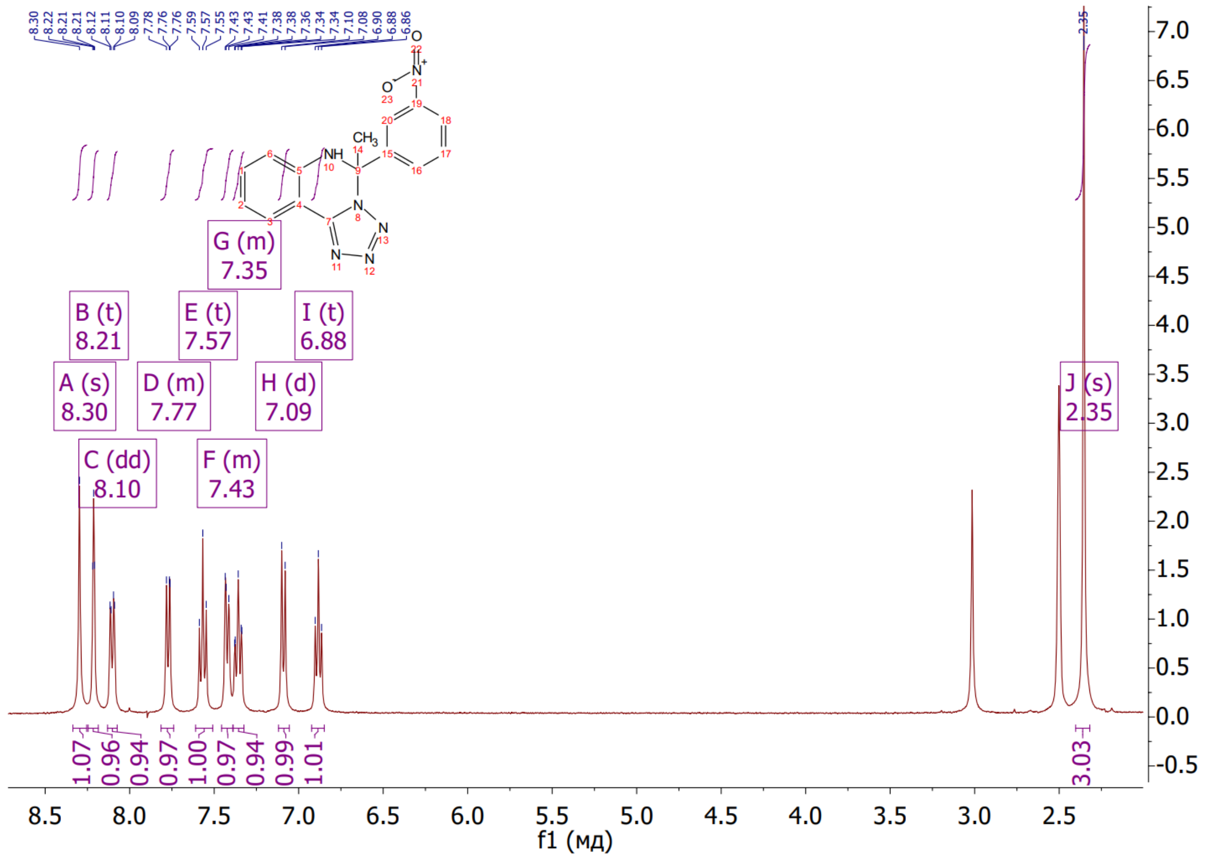

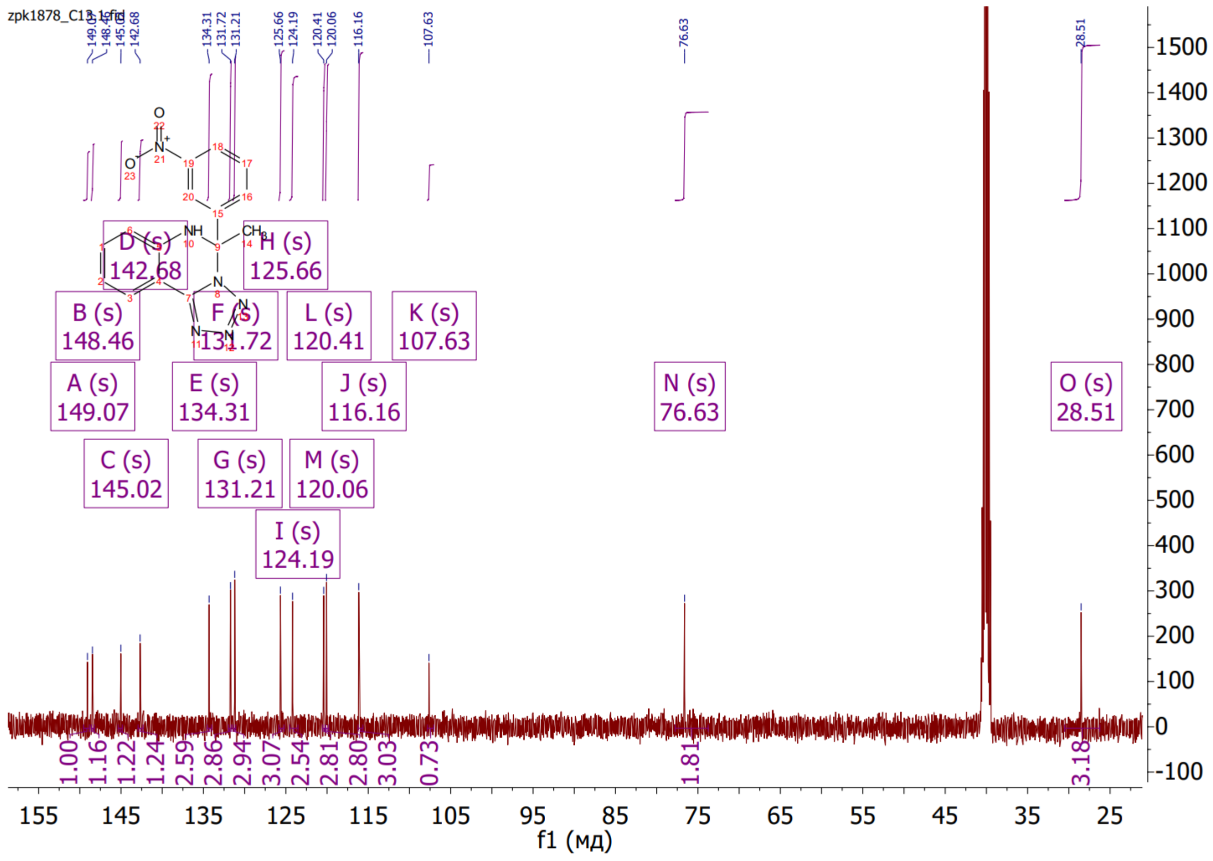

IR spectrum of **c12**.

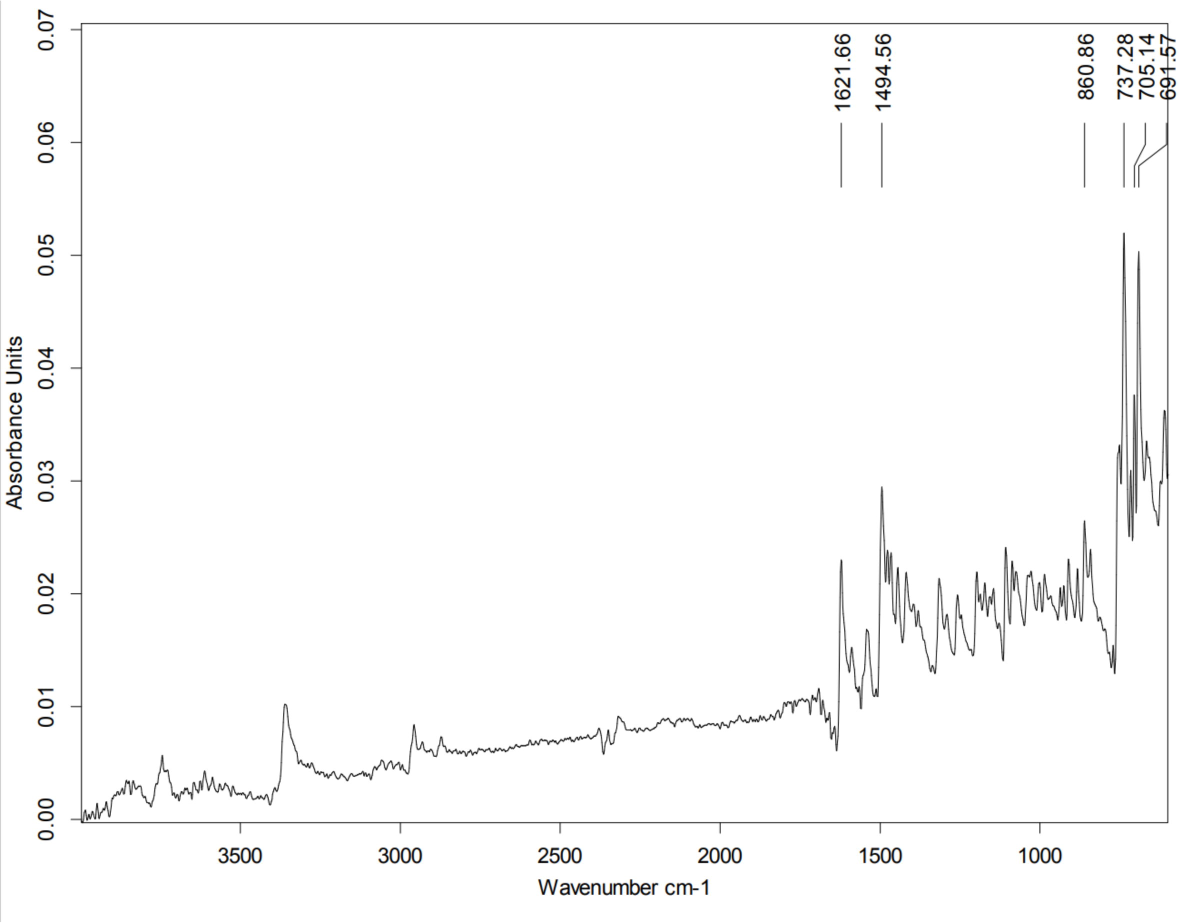

LC-MS spectrum of **c12**.

MaxPeak: 97.54%
Ret_Time: 1.396 min

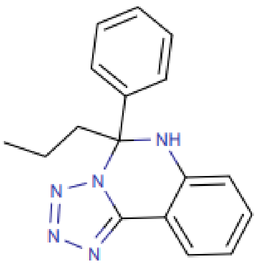

Mol Wt 291.35
Exact Mass 291.17

#	Time	Area%
1	1.310	2.46
2	1.396	97.54

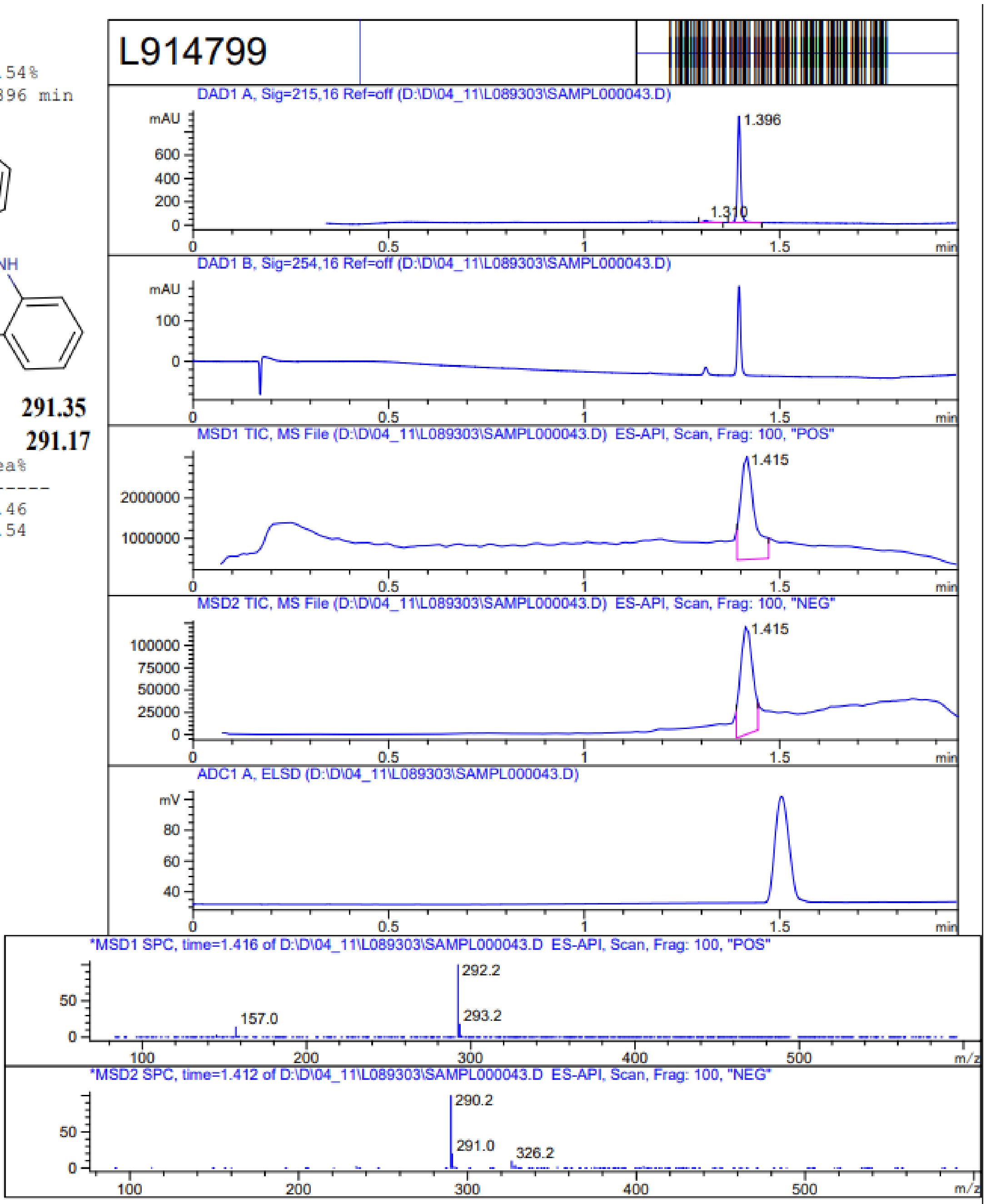

RT 1.415

RT 1.415

Inj.Date 4/11/2018 N P2-F-02 -3- Acq. Method C:\CHEM32\-> ->

^{1}H spectrum of **c12**.

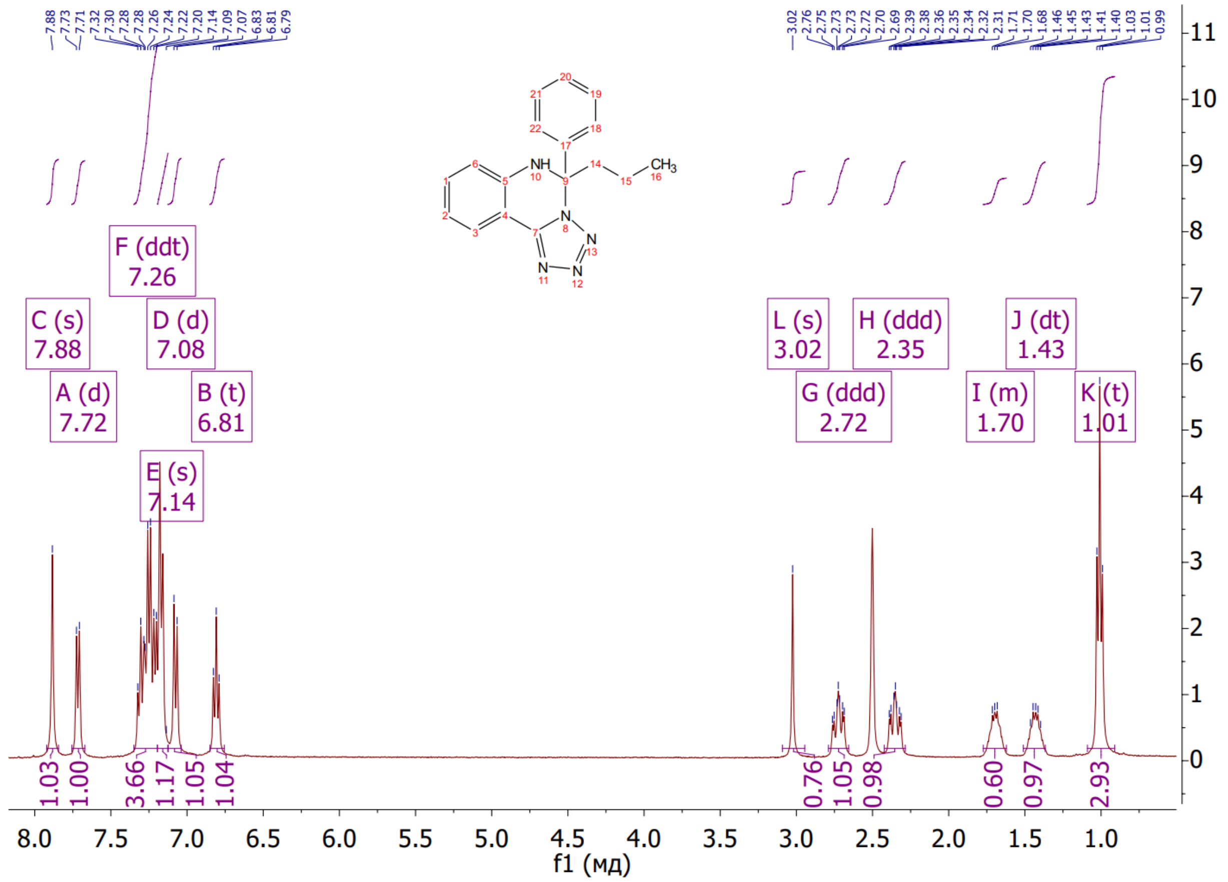

Table S1. CB-Dock2 website results of cavity detection and protein-ligand blind molecular docking.

PDB ID	ID	Vina Score, kkal/mol	Cavity Volume (Å3)	Center (X, Y, Z)	Docking Size (X, Y, Z)	Contact Residues
7YMU	c1	-8.1	1398	-34, -13, 60	19, 19, 19	Chain A: GLU153 ASP154 TRP156 GLU181 TRP184 GLU185 LEU187 ASN188 ILE266 GLY267 GLN268 ARG269 LYS318<br>Chain B: ASP154 TRP156 TRP184 GLU185 LEU187 ASN188 LYS191 LYS197 ILE266 GLY267 GLN268 ARG269 LYS318
	c5	-8.2	1106	-18, -18, 39	19, 19, 19	Chain B: SER13 GLY14 ALA15 SER16 GLY17 PHE18 ILE19 ALA20 SER37 VAL38 ARG39 VAL62 LYS63 ASP64 ILE65 ALA66 THR88 ALA89 SER90 PRO91 LEU92 ASN93 THR132 SER133 SER134 PHE135 ALA136 VAL169 TYR172 PHE173 LYS176 PRO203 VAL204 TYR205 VAL206 SER223 SER224 GLU225 CYS244<br>Chain C: THR95
3FWK	c1	-7.9	1438	48, 17, -3	19, 26, 19	Chain A: SER60 TYR61 ASN62 GLY63 GLY64 LYS65 ASP66 CYS67 VAL106 PHE107 ILE108 HIS110 ASP112 THR113 PHE114 LEU117 MET143 PHE147 ILE162 GLY163 ILE164 ASP168 PRO169 PHE170 TRP184 ARG189 GLN191 TYR216 SER222 LEU223 GLY224 ASN225 GLU228 GLU296 ARG297 ARG300
	c5	-7.9	1438	48, 17, -3	19, 26, 19	Chain A: SER60 ASN62 GLY63 GLY64 LYS65 ASP66 CYS67 PHE107 ILE108 HIS110 ASP112 THR113 LEU117 THR142 MET143 ALA144 ILE162 GLY163 ILE164 ASP168 PRO169 PHE170 LEU174 THR180 ASP181 TRP184 ARG189 GLN191 TYR216 THR221 SER222 LEU223 GLY224 ASN225 GLU228 GLU296 ARG297 ARG300
1EQP	c1	-9.6	869	32, 41, 60	19, 19, 28	Chain A: GLU27 TYR29 GLY143 PHE144 ASP145 ASN146 ARG150 ASP151 TYR153 GLU192 ASP227 PHE229 GLN230 HIS253 HIS254 TYR255 PHE258 GLU262 TRP277 GLU292 LEU304 ASN305 GLY306 ARG309 ARG312 TYR317 TRP363 TRP373
	c5	-9.7	869	32, 41, 60	19, 19, 28	Chain A: GLU27 TYR29 ASN142 GLY143 PHE144 ASP145 ASN146 ARG150 ASP151 TYR153 GLU192 LEU194 PRO196 PHE229 HIS253 HIS254 TYR255 PHE258 GLU262 GLU292 LEU304 ASN305 GLY306 ARG309 ARG312 TYR317 TRP373
5JLC	c1	-9.6	4955	-42, 73, -23	33, 31, 19	Chain A: ALA70 ILE71 TYR73 GLY74 LEU96 LEU97 ARG99 MET101 PHE114 TYR127 LEU130 THR131 PHE135 ILE140 TYR141 LEU148 LYS152 PHE237 PRO239 ILE240 PHE242 VAL243 LEU308 GLY311 VAL312 LEU313 MET314 GLY315 GLY316 HIS318 THR319 HIS379 PRO380 LEU381 HIS382 SER383 LEU384 PHE385 ARG386 PRO464 PHE465 GLY466 HIS470 ARG471 CYS472 ILE473 GLY474 PHE509 THR510 SER511 MET512 VAL513

	**c5**	-8.8	4955	-42, 73, -23	33, 31, 19	Chain A: ALA70 TYR73 GLY74 LEU96 LEU97 ARG99 MET101 TYR127 LEU130 THR131 PHE135 ILE140 TYR141 LEU148 PHE237 THR238 PRO239 ILE240 PHE242 VAL243 LEU308 GLY311 VAL312 MET314 GLY315 GLY316 GLN317 HIS318 THR319 LEU381 HIS382 SER383 LEU384 PHE385 HIS406 HIS470 ARG471 CYS472 ILE473 GLY474 PHE509 THR510 SER511 MET512 VAL513
**5TZ1**	**c1**	-10.2	4183	66, 35, 41	32, 28, 19	Chain B: TRP57 PHE58 ALA61 TYR64 GLY65 PRO68 LEU87 LEU88 PHE105 TYR118 LEU121 THR122 PHE126 ILE131 TYR132 PRO230 ILE231 PHE233 VAL234 GLY303 ILE304 GLY307 GLY308 THR311 MET374 PRO375 LEU376 HIS377 SER378 ILE379 PHE380 ARG381 PRO462 PHE463 GLY464 HIS468 CYS470 ILE471 GLY472 PHE475 TYR505 SER506 SER507 MET508
	**c5**	-9.2	1811	75, 51, 24	19, 19, 19	Chain A: VAL51 PHE52 TYR53 TRP54 ILE55 PHE58 GLY59 SER60 ALA61 ALA62 SER63 GLN66 GLN67 GLU70 PHE71 SER74 CYS75 LYS78<br>Chain B: PHE52 TRP54 ILE55 ALA62 SER63 GLU70 PHE71 SER74 LYS78
**7VRD**	**c1**	-8.1	5109	-10, -18, 24	35, 31, 35	Chain A: LYS221 THR222 TYR260 ASP261 LEU262 ASP263 SER269 ASP270 LYS273 TRP274 LEU275 PRO278 GLN279 ALA281 ASP282 LEU283 GLU285 GLN286 ILE288 SER289 GLU290 TYR291<br>Chain C: HIS45 GLU46 ALA47 LEU48 GLU49 LEU50 THR101 PRO102 ASN103 SER105
	**c5**	-8.1	5109	-10, -18, 24	35, 31, 35	Chain A: PRO223 ASP228 MET231 LYS240 ASP261 LEU262 LEU275 GLN279 LEU280 ASP282 LEU283 GLN286 LEU287 ILE288 SER289 GLU290 TYR291 PRO292 ILE293 VAL294 ASP317 LYS318 ILE319 LYS432 PHE434 GLN435<br>Chain C: THR101 PRO102 ASN103 SER105 SER252 TYR255 LYS256 ASP257 GLY258 TRP274 GLU302 ASP303 ASP304 TRP305 ASP306 ALA307 ASN330
**7O9Q**	**c1**	-9.4	2122	-4, 54, 61	25, 19, 19	Chain A: GLY101 PRO102 ASN129 GLU157 THR158 ASN159 ASP160 PRO161<br>Chain B: PHE210 TYR212 THR228 THR229 ALA230 ALA232 GLY249 ASN250 ILE251 ALA253 GLN294 GLU296 THR297 LEU298 SER299 THR307 LEU309 LEU311
	**c5**	-9.1	2122	-4, 54, 61	25, 19, 19	Chain A: GLY101 PRO102 SER103 ASN129 THR130 ASP131 GLU157 THR158 ASN159 ASP160 PRO161 LEU163<br>Chain B: PHE210 TYR212 THR228 THR229 ALA230 LEU231 ALA232 GLY249 ASN250 ILE251 ALA253 GLN294 GLU296 THR297 LEU298 SER299 LEU309 LEU311
**7EKU**	**c1**	-9.5	6320	12, 102, 175	34, 28, 35	Chain A: TYR23 THR29 PRO31 LEU33 ASN144 ARG604 HIS605 GLY606 GLY607 ARG608 PRO609 LYS614 PHE615 HIS641 SER642 VAL643 LYS644 CYS645 VAL646 LYS653 THR658 PRO659 PRO660 HIS661 ALA662 SER694 SER695 GLU745 VAL747 ASP748 ILE749 GLU750 ASP751 SER752 ALA770 LYS771 ASP875 ARG879
	**c5**	-9.2	3149	70, 121, 215	33, 28, 25	Chain B: LEU8 ARG10 LEU11 SER12 ASP13 PRO17 VAL18 THR19 SER20 LYS50 ALA52 ILE53 PRO54 ALA55 LYS88 GLU93 ASP94 ASP95 HIS96 PHE615 ASP619 ASP620 PHE621

						PRO622 PRO624 ASP626 VAL627 LYS628 MET650 ILE651 PRO652 ARG937 LYS938 PHE941 TYR942 LYS1027 SER1028 THR1029 VAL1030 PHE1031 ARG1479 GLU1483
**7VPR**	**c1**	-10.4	1450	-24, -18, -55	19, 26, 19	Chain D: ILE727 LEU731 TRP742 VAL746 VAL750 PHE833 ALA834 PRO836 MET858 ARG859 TYR861 TYR862 LEU865 THR869 LEU879 VAL882 LEU886 VAL890 GLY898 MET901 MET902 PHE905 LEU906
	**c5**	-7.9	1450	-24, -18, -55	19, 26, 19	Chain D: ILE727 LEU731 TRP742 VAL746 LYS747 VAL750 VAL832 PHE833 PRO836 MET858 ARG859 TYR861 TYR862 LEU865 THR869 LEU879 VAL882 VAL885 LEU886 PRO887 VAL890 GLY898 MET899 MET901 MET902 PHE905 LEU906
**7QP0**	**c1**	-7.6	1285	-8, -28, 22	19, 19, 29	Chain A: ASN106 ILE108 LEU114 ARG115 GLY116 ILE118 ASN119 HIS122 PHE125 PRO136 GLU137 ILE139 VAL140 MET141 LEU142 ASP145 GLN146 GLU148 VAL150 LYS151<br>Chain B: ASN106 ILE108 GLY109 ALA113 LEU114 ARG115 ILE118 ASN119 PHE125 ILE139 VAL140 MET141 THR143 ASP145 GLN146 GLU148 VAL150 LYS151
	**c5**	-7.8	1285	-8, -28, 22	19, 19, 29	Chain A: ASN106 ILE108 LEU114 ARG115 ILE118 ASN119 HIS122 PHE125 PRO136 ILE139 VAL140 MET141 THR143 ASP145 GLN146 VAL150 LYS151<br>Chain B: ASN106 ARG115 ILE118 ASN119 HIS122 PHE125 PRO136 GLU137 ILE139 VAL140 MET141 LEU142 ASP145 GLN146 GLU148 VAL150 LYS151 GLU156 ASN157 ILE158 ARG160 ALA161 TRP164
**7VPS**	**c1**	-9.8	2295	20, 6, 40	19, 32, 19	Chain A: ASN141 GLU142 ASN143 GLN144 GLN181 LEU182 TYR184 THR185 SER216 GLU219 PRO220 LYS258<br>Chain B: PHE225 ASN226 SER227 GLU228 VAL230 ILE233 ARG234 PRO261 THR262 ALA264 LYS265 LEU266 TYR268 SER269 PHE270 ASP271 THR274 ASP298 ALA299 ARG300 ILE301 LYS303 ARG304
	**c5**	-9.3	2295	20, 6, 40	19, 32, 19	Chain A: MET140 ASN141 GLU142 ASN143 GLN149 LEU178 ILE180 GLN181 LEU182 TYR184 THR185 CYS215 SER216 GLU219 PRO220 LYS258<br>Chain B: LEU222 PHE225 ASN226 SER227 GLU228 VAL230 ILE233 PRO261 THR262 ALA264 LYS265 LEU266 ILE267 TYR268 SER269 PHE270 ASP271 THR274 ASP298 ALA299 ARG300 ILE301 LYS303 ARG304
**7P43**	**c1**	-9.3	3039	28, 33, 30	29, 26, 19	Chain A: ILE451 LYS455 VAL492 GLY493 ASP494 LYS495 PHE499 TRP500 TRP555 ASP557 ARG560 GLY562 ASN563 ASN564 SER566 HIS568 TYR569<br>Chain B: ARG58 TYR61 TYR180 GLN181 PHE182 HIS184 GLN185 ARG186 PRO187 LYS188 LYS269 HIS276 SER277 MET278 GLY279 ILE347 ASP348 VAL349 GLN351 LEU410 SER413
	**c5**	-8.8	3039	28, 33, 30	29, 26, 19	Chain A: LEU491 VAL492 GLY493 TRP555 ASP557 ARG560 GLY562 ASN563 ASN564 SER566 HIS568 TYR569

						Chain B: TYR61 TYR180 GLN181 PHE182 HIS184 GLN185 ARG186 PRO187 LYS188 PHE189 ASN190 ASP194 ASP348 VAL349 GLN351 LEU409 LEU410 PRO411 SER413
**7VPT**	**c1**	-7.1	213	-18, 21, 1	19, 19, 19	Chain A: LEU116 SER117 ARG118 GLU119 PRO122 LEU150 GLU151 TRP154 THR157 ASN158 SER161 GLU189 GLU192 GLN193 TRP196 ASN200 LYS229 THR231 ARG234 THR235 TRP238 GLU273 ASP277
	**c5**	-7	213	-18, 21, 1	19, 19, 19	Chain A: LEU116 SER117 LEU150 GLU151 TRP154 THR157 ASN158 GLU189 GLU192 GLN193 TRP196 LYS229 THR231 THR235
**4D3W**	**c1**	-7.1	1054	-9, 15, 17	19, 19, 19	Chain A: ILE54 THR56 PRO57 LYS58 LYS59 SER62 LYS101 ASP103 TYR146 GLY147 TYR148 PHE177 ARG182 ASP183 PHE253 SER254 LEU255 ASP256 ASP257
	**c5**	-7.4	219	11, 13, 3	19, 19, 19	Chain A: LYS41 TYR83 LEU84 ASP85 PRO86 ASN87 PRO89 ARG90 PHE168 PHE189 GLY190 ASN191 TYR192 TYR195 ILE197
**4HOG**	**c1**	-8.5	8183	-1, -21, -16	35, 35, 35	Chain A: GLU16 ASN100 GLY123 GLY124 GLU125 ILE126 MET131 ASP132 PHE156 LEU157 GLN158 LYS159 GLN160 GLU161 GLN164 ARG165 LYS216 LYS217 LEU218 GLU219 HIS220 HIS222<br>Chain B: ASN100 GLY123 GLU125 ILE126 GLN129 ASP132 GLN164 ARG165 LYS216 LYS217 LEU218 GLU219 HIS220 HIS223 HIS224 HIS225
	**c5**	-7.9	8183	-1, -21, -16	35, 35, 35	Chain A: LYS3 VAL4 VAL6 ASN96 ARG99 ASN100 LEU102 THR103 GLN106 ARG128 ASP132 LEU133 ALA134 ASP135 HIS224 HIS225 HIS227<br>Chain B: VAL6 GLU16 GLY55 ARG56 LYS57 VAL77 SER78 ARG79 SER80 SER97 LEU98 ARG99 ASN100 LEU102 THR103 GLU125 ILE126 ARG128 GLN129 ASP132 LEU133 PHE156 LEU157 GLN158 LYS159 GLN160 GLU161
**4KQ6**	**c1**	-9.4	4930	7, 19, 74	31, 35, 19	Chain C: HIS24 ARG26 TRP27 ASN28 ARG29 VAL55 GLU56 THR57 PRO59 GLY60 SER61 PHE62 GLU63 VAL90 LEU91 ILE92 LYS93 GLY94 HIS98 PHE99 ILE102 Chain D: GLY5 LEU6 GLY7 GLN8 LEU9 ASP10 GLN11 ASN12 TYR13 ARG42 LEU46 PHE123 ARG137 MET146 HIS149 ASP152 TRP153 ALA155 ALA156 GLU159 LYS163
	**c5**	-9.4	4930	7, 19, 74	31, 35, 19	Chain C: ARG26 TRP27 ASN28 ARG29 VAL55 GLU56 THR57 PRO59 GLY60 SER61 PHE62 GLU63 VAL90 LEU91 ILE92 LYS93 GLY94 HIS98 PHE99 ILE102<br>Chain D: GLY5 LEU6 GLY7 GLN8 LEU9 ASP10 GLN11 ASN12 TYR13 ARG42 LEU46 ILE122 PHE123 ARG137 HIS147 HIS149 GLU151 ASP152 TRP153 ALA155 ALA156 GLU159 LYS163
**4N9N**	**c1**	-9.6	1686	-22, 4, 33	29, 19, 19	Chain B: ASN1002 GLU1005 ILE1009 THR1157 TRP1158 ASP1159 TYR1161 VAL724 ASP725 SER726 SER728 PRO729 SER730 TRP732 ILE733 PHE825 LEU829 PHE833 LEU837 ALA844 ARG846 ILE847 ARG849 TYR851 TYR852 ARG856 GLY871 VAL872 THR873 GLN874
	**c5**	-9.9	1686	-22, 4, 33	29, 19, 19	Chain B: ASN1002 ILE1003 GLU1005 LEU1007 ILE1009 THR1157 TRP1158 ASP1159 TYR1161 VAL724 ASP725 SER726 SER728 PRO729 SER730 TRP732 ILE733 LEU837 GLY840 ASP841

						LEU842 GLY843 ALA844 ARG846 ILE847 ARG849 TYR852 ARG856 GLY871 VAL872 THR873 GLN874
**2C1T**	**c1**	-9.2	1534	36, -31, 3	19, 19, 19	Chain A: GLU118 ARG120 PRO121 PRO122 ILE123 ASP124 ILE127 GLN128 SER160 GLY161 THR162 ALA164 GLN165 LYS167 VAL168 ASP171<br>Chain B: TRP195 ASN199 TRP237 VAL275 TRP279 THR313 LEU314 VAL315 THR317 PRO318 LYS354<br>Chain D: ARG12 GLU13 TYR15 ASP16 SER17 ASN18
	**c5**	-8.7	804	34, -7, 3	19, 19, 19	Chain A: ARG120 PRO121 ILE123 ASP124 ILE127 GLN128 VAL132 PRO133 SER160 GLY161 THR162 ALA164 GLN165 LYS167 VAL168 ASP171<br>Chain B: TRP237 VAL275 ASP276 TRP279 THR313 LEU314 THR317 PRO318 LYS354<br>Chain D: GLN11 ARG12 GLU13 THR14 TYR15 ASP16 SER17 ASN18

Table S2. Parameters of QSAR equation and their definition.

Parameter	Definition
n	the number of data points used to train the model
R^2	the coefficient of determination, indicating the proportion of the variance in the dependent variable (biological activity) that is predictable from the independent variables (descriptors)
Mor26m, Mor29e	3D-MoRSE descriptors, which are numerical representations of molecular structure characteristics. These descriptors are weighted by mass and Sanderson electronegativity
s	denotes the standard error of the estimate
F	F-statistic, which assesses the overall significance of the model
p	p-value associated with the F-statistic, indicating the probability of observing the data given that the null hypothesis (the model has no predictive value) is true
RMSEtr	the root mean square error of the training set, measuring the average deviation between predicted and observed values in the training data
R^2-R^2cv	the cross-validated coefficient of determination, indicating the predictive ability of the model on unseen data
RMSEcv	the root mean square error of cross-validation, measuring the predictive accuracy of the model
MAEcv	the mean absolute error of cross-validation
PRESScv	the prediction error sum of squares for cross-validation
CCCcv	the concordance correlation coefficient for cross-validation
RMSEex	the root mean square error of external validation, measuring the predictive accuracy of the model on independent test data
MAEex	the mean absolute error of external validation
PRESSext	the prediction error sum of squares for external validation

Table S3. SMILES of studied substances.

#	SMILES
**a1**	C12=CC=CC=C1NC3(CCCC3)N4C2=NN=N4
**a2**	C12=CC=CC=C1NC3(CCCCC3)N4C2=NN=N4
**b1**	O=C1C2(C(C=CC=C3)=C3N1)N4C(C5=CC=CC=C5N2)=NN=N4
**b2**	O=C1N(CC(NC2=CC=CC(Cl)=C2)=O)C3=C(C=CC=C3)C41N5C(C6=CC=CC=C6N4)=NN=N5
**b3**	O=C1C2(C(C=CC=C3)=C3N1CC(NCC4=CC=CC=C4C(F)(F)F)=O)N5C(C6=CC=CC=C6N2)=NN=N5
**c1**	CC(C=C1)=CC=C1C2N3C(C4=CC=CC=C4N2)=NN=N3
**c2**	ClC(C=C1)=CC=C1C2N3C(C4=CC=CC=C4N2)=NN=N3
**c3**	CC1(C2=CC=CC=C2)N3C(C4=CC=CC=C4N1)=NN=N3
**c4**	CC1(C2=C(Cl)C=CC=C2)N3C(C4=CC=CC=C4N1)=NN=N3
**c5**	CC1(C2=CC=C(Br)C=C2)N3C(C4=CC=CC=C4N1)=NN=N3
**c6**	CC1(C2=CC=C(O)C=C2)N3C(C4=CC=CC=C4N1)=NN=N3
**c7**	CC1(C2=CC=C(C#N)C=C2)N3C(C4=CC=CC=C4N1)=NN=N3
**c8**	CC1(C2=CC=C(C=C2)[N+]([O-])=O)N3C(C4=CC=CC=C4N1)=NN=N3
**c9**	CC1(C2=CC=CC(C(O)=O)=C2)N3C(C4=CC=CC=C4N1)=NN=N3
**c10**	CC1(C2=CC=C(C(O)=O)C=C2)N3C(C4=CC=CC=C4N1)=NN=N3
**c11**	O=[N+](C1=CC(C2(C)NC3=C(C=CC=C3)C4=NN=NN24)=CC=C1)[O-]
**c12**	CCCC1(C2=CC=CC=C2)NC3=C(C=CC=C3)C4=NN=NN14
**d1**	CC1(C2=NC=CC=C2)N3C(C4=CC=CC=C4N1)=NN=N3
**d2**	CC1(C2=CC=CN=C2)N3C(C4=CC=CC=C4N1)=NN=N3
**d3**	CC1(C2=CC=NC=C2)N3C(C4=CC=CC=C4N1)=NN=N3

Table S4. Predicted herbicide, environmental and human toxicity by CropCSM of Biosig Lab.

#	*Herbicide Activity*	*Environmental Toxicity* Honey Bee Toxicity	Avian Toxicity	Minnow Toxicity (log mg/kg/day)	*Human Toxicity* AMES Toxicity	Rat Acute Toxicity (LD_{50}, mg/kg)	Rat Chronic Toxicity (LOAEL, mg/kg/day)
**a1**	No	No	Yes	1.37	Yes	494.9	11.9
**a2**	No	No	Yes	1.39	Yes	487.1	10.7
**b1**	No	No	No	0.85	Yes	497.7	32.0
**b2**	No	No	No	0.04	No	1465.9	38.6
**b3**	No	No	No	-0.06	No	681.8	51.9
**c1**	No	No	No	0.45	Yes	749.1	13.5
**c2**	No	No	No	0.15	Yes	770.2	23.7
**c3**	No	No	No	0.59	Yes	812.3	14.0
**c4**	No	No	No	0.07	Yes	740.9	23.5
**c5**	No	No	No	0.03	Yes	959.2	20.3
**c6**	No	No	No	0.71	Yes	647.9	17.9
**c7**	No	No	No	0.77	Yes	764.8	17.9
**c8**	No	No	No	0.59	Yes	742.6	25.9
**c9**	No	No	No	0.63	Yes	899.0	18.7
**c10**	No	No	No	0.63	Yes	855.7	20.3
**c11**	No	No	No	0.68	Yes	743.1	35.3
**c12**	No	No	No	0.30	Yes	729.2	13.0
**d1**	No	No	No	1.58	Yes	789.1	13.9
**d2**	No	No	No	1.55	Yes	695.7	15.8
**d3**	No	No	No	1.58	Yes	692.1	14.3

Calculated minnow toxicity (log LC_{50} mg/kg/day, results below -0.3: high acute), rat acute toxicity (LD_{50}, mg/kg; results under 50: strong; 50-500: moderate; 500-5000: slightly; over 5000: safe), and rat chronic toxicity (LOAEL, mg/kg/day; results under 10: strong; 10-50: medium; over 50: weak).

Table S5. Pearson correlation results calculated in Origin 2018.

Values	Parameter	MT*	RAT	RCT	CYP51	MIC
**MT***	Pearson Corr.	1	-0.48236	-0.55494	0.72534	0.0448
	p-value	-	0.03124	0.01109	6.57958E-4	0.92402
**RAT**	Pearson Corr.	-0.48236	1	0.2968	-0.51301	-0.37505
	p-value	0.03124	-	0.20382	0.02947	0.40712
**RCT**	Pearson Corr.	-0.55494	0.2968	1	-0.87526	-0.03262
	p-value	0.01109	0.20382	-	1.97927E-6	0.94465
**CYP51**	Pearson Corr.	0.72534	-0.51301	-0.87526	1	0.33929
	p-value	6.57958E-4	0.02947	1.97927E-6		0.51059
**MIC**	Pearson Corr.	0.0448	-0.37505	-0.03262	0.33929	1
	p-value	0.92402	0.40712	0.94465	0.51059	--

MT: Minnow Toxicity (log LD_{50}, mg/kg/day), RAT: Rat Acute Toxicity (LD_{50}, mg/kg), RCT: Rat Chronic Toxicity (LOAEL, mg/kg/day), CYP51: affinity to CYP51, PDB ID 5tz1 (kcal/mol), MIC: minimum inhibition concentration against *C. glabrata*, μM.
